# Epigenetic signatures relating to disease-associated genotypic burden in familial risk of bipolar disorder

**DOI:** 10.1038/s41398-022-02079-6

**Published:** 2022-08-03

**Authors:** Sonia Hesam-Shariati, Bronwyn J. Overs, Gloria Roberts, Claudio Toma, Oliver J. Watkeys, Melissa J. Green, Kerrie D. Pierce, Howard J. Edenberg, Holly C. Wilcox, Emma K. Stapp, Melvin G. McInnis, Leslie A. Hulvershorn, John I. Nurnberger, Peter R. Schofield, Philip B. Mitchell, Janice M. Fullerton

**Affiliations:** 1 Neuroscience Research Australia, Sydney, NSW Australia; 2 School of Medical Sciences, Faculty of Medicine, University of New South Wales, Sydney, NSW Australia; 3 School of Psychiatry, Faculty of Medicine, University of New South Wales, Sydney, NSW Australia; 4 Centro de Biología Molecular “Severo Ochoa”, Universidad Autónoma de Madrid/CSIC, Madrid, Spain; 5 Department of Medical and Molecular Genetics, Indiana University, Indianapolis, IN USA; 6 Department of Biochemistry and Molecular Biology, Indiana University, Indianapolis, IN USA; 7 Child Psychiatry & Public Health, Johns Hopkins University, Baltimore, MD USA; 8 Johns Hopkins Bloomberg School of Public Health, Baltimore, MD USA; 9 National Institute of Mental Health, Bethesda, MD USA; 10 Department of Psychiatry, University of Michigan, Ann Arbor, MI USA; 11 Department of Psychiatry, Stark Neurosciences Research Institute, Indiana University School of Medicine, Indianapolis, IN USA

**Keywords:** Comparative genomics, Bipolar disorder, Predictive markers, Epigenetics and behaviour

## Abstract

Environmental factors contribute to risk of bipolar disorder (BD), but how environmental factors impact the development of psychopathology within the context of elevated genetic risk is unknown. We herein sought to identify epigenetic signatures operating in the context of polygenic risk for BD in young people at high familial risk (HR) of BD. Peripheral blood-derived DNA was assayed using Illumina PsychArray, and Methylation-450K or -EPIC BeadChips. Polygenic risk scores (PRS) were calculated using summary statistics from recent genome-wide association studies for BD, major depressive disorder (MDD) and cross-disorder (meta-analysis of eight psychiatric disorders). Unrelated HR participants of European ancestry (*n* = 103) were stratified based on their BD-PRS score within the HR-population distribution, and the top two quintiles (High-BD-PRS; *n* = 41) compared against the bottom two quintiles (Low-BD-PRS; *n* = 41). The High-BD-PRS stratum also had higher mean cross-disorder-PRS and MDD-PRS (ANCOVA *p* = 0.035 and *p* = 0.024, respectively). We evaluated DNA methylation differences between High-BD-PRS and Low-BD-PRS strata using linear models. One differentially methylated probe (DMP) (cg00933603; *p* = 3.54 × 10^−7^) in *VARS2*, a mitochondrial aminoacyl-tRNA synthetase, remained significantly hypomethylated after multiple-testing correction. Overall, BD-PRS appeared to broadly impact epigenetic processes, with 1,183 genes mapped to nominal DMPs (*p* < 0.05); these displayed convergence with genes previously associated with BD, schizophrenia, chronotype, and risk taking. We tested poly-methylomic epigenetic profiles derived from nominal DMPs in two independent samples (*n* = 54 and *n* = 82, respectively), and conducted an exploratory evaluation of the effects of family environment, indexing cohesion and flexibility. This study highlights an important interplay between heritable risk and epigenetic factors, which warrant further exploration.

## Introduction

Bipolar disorder (BD) is a highly heritable mental illness, for which genetic factors explain ~60–85% of risk variance [1]; the remaining variance is explained by non-genetic factors, including environmental contributors. First-degree relatives of probands with BD have 5–10-fold increased risk of developing BD themselves [1–3], and are at increased risk of broader psychopathology [4, 5], including major depression, anxiety, behavioural and substance use disorders; therefore, young first-degree relatives of those with BD are considered high risk (HR) for later mental illness. The elucidation of clinical antecedents of the BD prodrome is an active research area [6–10], but specific precursors are heterogeneous, and biomarkers of risk trajectories are a research priority.

Genome-wide association studies (GWAS) have identified many single nucleotide polymorphisms (SNPs) associated with increased disease risk, each with small individual effect [11–13]. Many disease-associated SNPs are shared amongst psychiatric disorders, with substantial genetic correlation between BD and schizophrenia, and BD and major depressive disorder (MDD) [13–17]. GWAS have demonstrated the polygenic nature of BD, where common SNPs identified to date collectively account for ~25% of the estimated heritability [12–15]. Indeed, individuals with polygenic risk scores (PRS) at the extremes of a population distribution have substantially altered risk of developing a psychiatric disorder—those in the top 10% of the BD-PRS distribution have an odds ratio of 9.3 of developing BD compared to the lowest decile [13]—yet the predictive capacity of PRS is currently limited [18], with inadequate sensitivity and specificity as PRS will only capture *part* of the genetic contribution [19]. SNP-based heritability falls short of heritability estimates from family, twin and epidemiologic studies; [20] the latter approaches typically employ models that include additive genetic and common/unique environmental factors (and their interaction), whereas SNP-based heritability estimates typically only model additive genetic variance [20]. Moreover, some of the “missing heritability” may also reside in rare variants not captured by GWAS [21–23], and/or relate to other familial factors that are not encoded in nucleotide-level DNA sequence variation.

Family history is the strongest current predictor of future BD [7] and is sometimes posited as a proxy for genetic transmission, but likely reflects dynamic gene-environment interplay from preconception through the life span. Twin studies also indicate environmental contribution to BD [24]. Several environmental risk factors have been posited—including prenatal infections, childhood maltreatment, and psychological stress [25]—but the attributable impact of such factors is small and not disease-specific. Families that include a parent with BD have lower parent-reported cohesion compared to families with no parental psychiatric disorders [26], and while offspring-centred reports are less common, child reports of lower cohesion and adaptability, and higher conflict environments are also noted [27]; these factors potentially confound family history with environmental elements. Furthermore, children who experienced early maternal loss have a four-fold increased risk of BD [28], suggesting that early trauma and altered home-environment dynamics may influence disease trajectory. Indeed, children are influenced by both genes and environment provided by parents, which cannot be easily disambiguated to apportion causation [29]. Moreover, environmental effects are likely conditional on genetic factors, which are only partially appraised by existing gene-environment studies [30]. Also, genetic nurture may include indirect genetic effects from parental genes that are not transmitted [31]. Thus, there is increasing interest in the role of specific gene-environment interactions, as well as the mechanisms by which risk factors interact in the development of BD [32].

Environmental factors can potentially impact gene expression through epigenetic modulations [33]. One of the most studied epigenetic processes is DNA methylation; the addition of a methyl group to the 5′ cytosine of a cytosine-guanine sequence (CpG) [33]. To date, there are no large-scale epigenome-wide studies in BD [34], although several candidate-gene epigenetic studies [35, 36] and pharmaco-epigenomic studies of antipsychotic medicines [37] have been performed. Furthermore, GWAS signals for schizophrenia—a condition that shares genetic overlap with BD [17, 38]—are enriched in human-specific methylated regions [39], implying mechanistic overlap between genetic and epigenetic risk. Interestingly, a recent epigenetic element-based transcriptome-wide association study identified genes that contribute to BD heritability beyond those explained by GWAS-associated SNPs [40], suggesting that epigenetic regulation may further contribute to heritability. Epigenetic factors may even contribute to transgenerational genomic regulation [41], although this mechanism of genomic transmission in humans remains controversial [42].

To identify biomarkers for illness-onset, a prospective longitudinal approach is required. One small prospective study that compared HR participants who developed BD or MDD (*n* = 22) to those who remained well (*n* = 23) identified 22,543 nominally differentially methylated CpGs (*p* < 0.05) [43], although no probe passed epigenome-wide correction for multiple testing. To date, the characterisation of methylation differences relating to polygenic burden in individuals with familial risk for BD has not been performed. Thus, we undertook the identification of differentially methylated positions (DMPs) in young HR participants, stratified by genetic burden of common BD-associated risk alleles, to characterise epigenetic modifications operating beyond polygenic additive risk. Methylation signatures derived from the cumulative effect of multiple CpGs were then validated in independent samples of BD cases, HR, and controls. Finally, the impact of family environment measures on methylation signatures were explored, to further understand relationships between familial and environmental risk factors associated with the development of psychopathology.

## Materials and methods

**Fig. 1:**
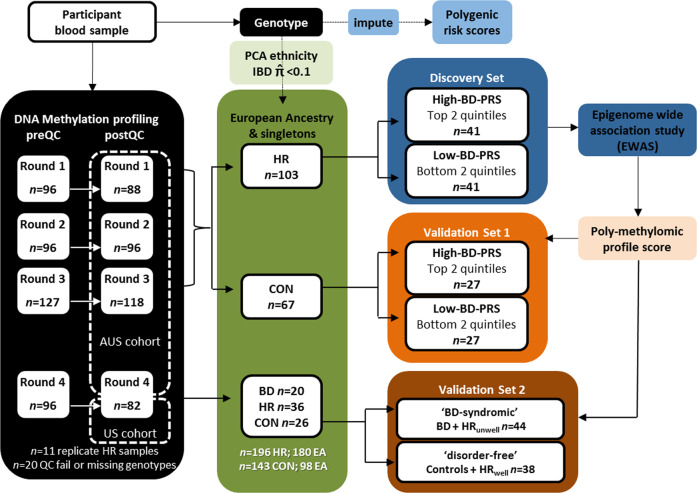
Schematic overview of methods and the derivation of discovery and validation samples. PRS polygenic risk score, PMPS poly-methylomic profile score, HR high-risk individuals, CON control, BD bipolar disorder, HR_unwell_ high-risk individuals who developed sub-threshold or threshold BD at baseline or follow-up, HR_well_ high-risk individuals who remained well after follow-up, EA European ancestry based on components from multidimensional scaling analysis of SNP genotype data.

An overview of the approach is summarised in Fig. 1.

### Study participants

Australian participants were aged 12–30 years and recruited as previously described [44, 45]. Briefly, HR and BD participants were recruited from families who had previously participated in BD family studies, specialised BD research clinics, mental health consumer organisations, or response to public notices. BD cases met DSM-IV criteria for BD type-I (BD-I) or type-II (BD-II). HR participants were the children or siblings of individuals with DSM-IV diagnoses of BD-I, BD-II, or schizoaffective disorder–bipolar-type (SABP), and did not personally have threshold diagnoses of these conditions at baseline (participants with sub-threshold BD “not otherwise specified” (BD-NOS) were not excluded). Control participants (CON) were recruited via print/electronic media, and noticeboards in universities and local communities. CON had no personal or familial (first-degree) history of BD-I, BD-II, recurrent unipolar disorder, SABP, schizophrenia, recurrent substance abuse or psychiatric hospitalisation, and no second-degree relative with a past mood-disorder hospitalisation or history of psychosis. Written informed consent was obtained from all participants, with additional parental consent for participants aged <16 years. This study was approved by University of New South Wales Human Research Ethics Committee (HREC Protocol 09/097).

High-risk participants from the United States were aged 12–21 years, and recruited from Indiana University, University of Michigan, Johns Hopkins University, and Washington University in St. Louis (with site-specific IRB approval), as previously described [46]. The US sites used recruitment criteria and clinical assessments identical to those used at the Australian site.

### Clinical assessments

Structured interviews were administered by staff with extensive clinical background and after specific training in each instrument, which comprised a battery of structured clinical interviews, self-report questionnaires and clinician rated assessments. *The Family Interview for Genetic Studies* (FIGS) [47] was administered to each participant or a member of their family to determine family history of mood or psychotic disorder. To determine psychiatric diagnoses for participants aged 12–21, both participants and their parent/s completed the *Kiddie-Schedule for Affective Disorders and Schizophrenia for School-Aged Children–Present and Lifetime Version* (K-SADS-BP; v2, July 2009) [46, 48]. To determine psychiatric diagnoses for participants aged 22-30 (including BD-probands), the *Diagnostic Interview for Genetic Studies* (DIGS; v4.0/BP, July 2005) [49] was administered. Consensus DSM-IV diagnoses were determined by two clinicians (i.e., psychiatrists with child specialty training, clinical psychologists, or clinical social workers) using best-estimate methodology [50], using the K-SADS or DIGS, FIGS, and medical records (where available). Functional capacity was determined via *Global Assessment of Functioning* (GAF) [51] or *Clinical Global Impression* (CGI) [52] rating scales.

A subset of participants completed the Family Adaptability and Cohesion Evaluation Scales (FACES-II) [53], a 30-item questionnaire from which a total score across the adaptability and cohesion subscales was used to represent family environment (described in Supplementary Material).

One to four follow-up clinical interviews were performed on HR and control participants to identify emergent psychopathology. Diagnoses were assigned a confidence rating on a 4-point scale; only those that met full DSM-IV criteria received a confidence rating ≥3. As previously described by Frankland et al. [8], participants with a best-estimate diagnosis of BD were categorised as either threshold converters (i.e. with BD-I or BD-II; confidence level 3–4) or sub-threshold converters (i.e. with BD-NOS; confidence level of 1–2); the latter diagnosis being made when participants did not meet the minimum 4 day duration criterion, but otherwise met full symptom criteria for hypomania. Participants (HR or control) reaching threshold or sub-threshold criteria at follow-up assessment for diagnosis of BD-I, BD-II, BD-NOS, with a confidence of ≥2 were considered “BD-syndromic”, and those with no clinical diagnoses after follow-up were considered “disorder-free”.

### Genotyping and polygenic risk scores

Peripheral blood samples were collected, DNA extracted, and genotyping performed on PsychArray-24 BeadChip as previously described [54] (details in Supplementary methods). An independent sample of BD cases (*n* = 264; described in [13] as “neuc1”) and controls (*n* = 1115) [55] were employed to infer optimal *p* value threshold for generation of PRS (*p*_T_; Supplementary Methods, Fig. S1).

Polygenic risk scores (PRS) were generated using summary statistics from the PGC GWAS for BD (*n* = 31,358 controls and *n* = 20,352 cases; excluding 6,201 participants from GAIN, BMAU, FAT2, MICH datasets which contained parents of some of the study participants) [12]. Data for MDD (*n* = 561,190 controls and *n* = 246,363 cases) [56] and cross-disorder (*n* = 494,162 controls and *n* = 232,964 cases with anorexia nervosa, attention-deficit/hyperactivity disorder, autism spectrum disorder, BD, MDD, obsessive-compulsive disorder, schizophrenia, and Tourette syndrome) [17] were also used. PRS were calculated in a single analysis for all participants simultaneously using PRSice v2.2.13 [57], employing unrelated individuals (identity-by-descent pi_hat < 0.1; Supplementary Methods) identified as European-ancestry by multidimensional scaling analysis (Supplementary Methods), and comparing BD cases to controls to define optimal *p*_T_ to explain the largest variance (Nagelkerke pseudo-*R*^2^); phenotypes for HR were coded unknown/missing (-9). Polygenic scoring employed linkage disequilibrium clumping, removal of strand-ambiguous SNPs, and SNPs with MAF < 0.05 or low imputation quality (INFO < 0.8), prior to PRS computation and use in subsequent analyses.

### Demographic comparisons

Group differences were examined using univariate general linear ANCOVA models, using SPSS Statistics for Windows v26 (IBM corp., Armonk, NY). For PRS group level comparisons in the extended cohort, generalised estimation equations (GEE) were employed to account for relationships within families which contained multiple relatives, and included genotype-derived MDS components (C1 and 2) as covariates.

### Methylation analysis

#### Epigenome-wide methylation profiling

Methylation quantification was performed on DNA derived from peripheral blood in four batches: batches 1 and 2 (both *n* = 96) employed the Illumina HumanMethylation450K array, and batches 3 and 4 (*n* = 127 and 96, respectively) employed the Illumina MethylEPIC BeadChip. The R package *meffil* [58] was used to extract signal intensities and initial quality control (QC) within each batch (Supplementary Material). The R package *Shinymethyl* [59] was used to conduct principal component analysis and visualise outliers within each batch. After removal of 11 technical replicates and 20 samples failing QC procedures, 384 participants remained (round 1 *n* = 88, round 2 *n* = 96, round 3 *n* = 118, round 4 *n* = 82) – 425,453 CpG probes that passed QC and were represented on both 450k and EPIC chips then underwent further filtering for EWAS as outlined below. To avoid potential bias in PRS due to ethnicity [60], the cohort was restricted to unrelated European-ancestry participants (Fig. S2), which were divided into Discovery and Validation sets (described below and in Fig. 1). Normalisation of methylation *β* values employed *meffil*, including technical covariates (sentrix position and sentrix group; the latter unique across batches) [58]. Normalisation of batches 1–3 was performed simultaneously across all three batches, and formed the Discovery Set and Validation Set 1, whereas batch 4 was normalised separately as Validation Set 2.

#### Tissue source, smoking status and cell count estimates

DNA was derived from three peripheral blood sources: whole blood, ficoll/buffy-coat, and isolated lymphoblasts (details in Supplementary Material). To account for inter-individual differences in cellular proportions, estimation of six cell types (B cells, CD4T, CD8T, granulocytes, monocytes and natural killer) was performed using *meffil’s* gse35069 profile [58]. Tissue source and cell count estimates (Fig. S3) were included as covariates, as described below.

Lifetime tobacco use collected via KSADS was available for ~60% of participants in this study, and current smoking status was available for ~16% (*n* = 36 out of 218 individuals; *n* = 5 current users, all in Validation Set 2). To predict smoking status in all participants, normalised *β* values at cg05575921 were used (Fig. S4), where *β* < 0.75 were classified as probable smokers [61].

#### Discovery set: HR youth stratified by BD-PRS

**Fig. 2:**
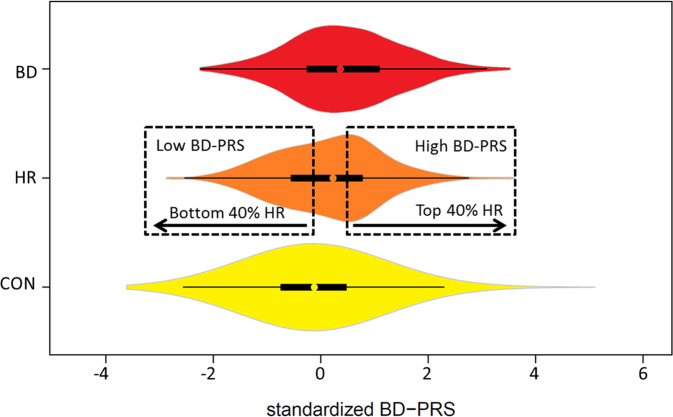
Violin plot of BD-PRS by clinical group in European-ancestry individuals. BD-PRS was standardised using the mean and standard deviation of all participants with genotype data (including an independent sample of BD cases and controls) regardless of availability of methylation data (*n* = 1699 controls, 354 HR, 355 BD; *M* = 0.0077695, SD = 7.543 × 10^−5^). The boxes and whiskers inside the violins indicate the 25–75th percentiles, and 1.5 times the interquartile range, respectively. The dashed rectangles indicate the stratification of HR participants into High-BD-PRS vs. Low-BD-PRS strata, as defined by the top and bottom two quintiles (i.e. 40% of the HR distribution), for the epigenome-wide association study. HR high risk, BD bipolar disorder, CON control, PRS polygenic risk score.

To stratify HR participants on the basis of their personal burden of BD-associated SNP variants, the distribution of BD-PRS within the HR sample was divided into quintiles; this stratification was undertaken on the basis that individuals with PRS in the extremes of a population distribution have substantially altered risk of developing a psychiatric disorder [13]. Quintiles were chosen to balance maximisation of the available sample, with exclusion of individuals with intermediate scores reflecting ‘average’ SNP burden. Thus two comparator groups were defined: the top two quintiles (40% of participants) formed the High-BD-PRS group (*n* = 41) and the bottom two quintiles (40% of participants) formed the Low-BD-PRS group (*n* = 41) (Table S1, Fig. 2).

#### Discovery EWAS: high vs. low-BD-PRS within HR youth

##### CpG selection

Probes were restricted to those which were: (1) variable in our data (*n* = 249,436)—defined by >5% variability in the difference between the 10th to 90th percentile range of *β* values across batches 1–3 [62], and (2) reported to be variable in blood (*n* = 244,724), as defined by Edgar et al. [63], and 3) correlated between blood and brain (*n* = 42,364; with mean r ≥ ±0.3 across prefrontal cortex, superior temporal gyrus and entorhinal cortex) as defined by Hannon et al. [62]. Restricting the discovery EWAS to variable blood-brain correlated probes had benefits in: (1) reducing the EWAS search space, and (2) focusing on CpGs that are likely to be functionally relevant in the primary disease-affected tissue. Importantly, ‘probe SNPs’ as annotated in the Illumina manifest file were removed prior to EWAS, to exclude direct effects of sequence variation out to 50 bp (*n* = 6,455). The final probe set included 35,907 CpGs, and *β* values for each probe were transformed into M-values using the ‘beta2m’ function in R package *lumi* [64].

Surrogate variable analyses: To identify and account for residual unmeasured variation that was not corrected by normalisation of technical covariates (sentrix position/chip/batch), the *‘be’* method in R package *SVA* [65] was used to identify surrogate variables considering BD-PRS stratum as the variable of interest (Low-BD-PRS = 0, High-BD-PRS = 1). Four significant surrogate variables (*p* < 0.05) were identified and included in downstream analysis.

EWAS: DMPs were identified using the R package *limma* [66], employing a linear regression model, with normalised M-values as outcome and BD-PRS stratum as predictor (Low-BD-PRS = 0, High-BD-PRS = 1). Covariates included sex, age, two ethnicity components (MDS C1 and C2; Supplementary Material), tissue source, blood-cell count estimates and four surrogate variables.

##### Bias correction

The R package *bacon* [67] was applied to correct test-statistic inflation, to minimise shift in distribution of effect sizes and risk of false-positive findings. Empirical null estimates were generated using a Gibbs Sampling algorithm in a Bayesian framework, based on 5000 iterations with burn-in period of 2000. DMPs whose inflation- and bias-corrected *p* values exceeded *p* < 1.39 × 10^−6^ (Bonferroni adjustment for 35,907 tests at *α* = 0.05) were considered epigenome-wide significant.

### Pathway analyses

DMPs at two *p* value thresholds (the Q–Q plot inflection point of *p* < 0.002, and *p* < 0.05; uncorrected) were mapped to genes using two complementary methods: (1) physical mapping of probes based on UCSC hg19 base-pair coordinates (as per Illumina manifest file), and (2) functional mapping of cis-regulatory regions using the *Genomic Regions Enrichment of Annotations Tool* (GREAT; v4.0.4) [68]. GREAT enables functional mapping of DMPs in non-coding regulatory regions (i.e. outside coding-sequence boundaries)—we employed *‘basal plus extension’* mode that includes genes 5 kb upstream, 1 kb downstream, plus 5 kb distal binding. The combined output of both methods created a gene list for pathway interrogation.

Finally, *Functional Mapping and Annotation of Genome-Wide Association Studies* (FUMA) [69] was employed with default parameters (i.e. not excluding MHC region) to examine enrichment of genes harbouring DMPs in biological function and pathway categories, and enrichment of gene expression across tissues [70]. Enrichments were considered significant if false discovery rate (FDR) *q* ≤ 0.05.

### Supplementary EWAS: post-hoc sensitivity analysis for interpretation of DMP functional enrichments

As the discovery EWAS employed a restricted set of 35,907 CpG probes that were blood-brain correlated [62], we reasoned that probe pre-selection might bias functional enrichments in genes mapped to DMPs. Therefore, a supplementary EWAS was conducted using 214,352 probes that passed QC and were variable in blood [63] and variable in our data (but without applying the blood-brain correlation filter), employing identical procedures as the primary EWAS. The multiple-testing correction threshold was adjusted to *p* < 2.33 × 10^−7^ for 214,352 tests at *α* = 0.05 [71].

### Validation using poly-methylomic profile scores

A ‘poly-methylomic profile score’ (PMPS) is a quantitative metric reflecting the degree of methylation at multiple sites across the epigenome (similar to calculating a PRS from GWAS summary statistics for *genotypic* data), as previously described [72]. A PMPS was calculated for each participant using effect sizes and *p*-values for DMPs from the discovery PRS-stratified EWAS, and applied to two independent validation sets (Fig. 1), as described below. Normality was assessed via Shapiro-Wilk tests in SPSS, and effect sizes reported as partial eta squared (η^2^p).

### Validation Set 1

To determine whether PMPS provided a replicable index of BD-PRS, we employed 67 *control* participants [European-ancestry, unrelated (pi_hat < 0.1)] derived from batch 1–3, thereby minimising technical variability between the Discovery Set (who were all HR) and Validation Set 1. As controls have an overlapping BD-PRS distribution to both BD and HR (Fig. 2), we reasoned that methylation changes that reflect BD-PRS variability should also be observable in controls.

Controls were divided into two strata based on their BD-PRS distribution: the top two quintiles (High-BD-PRS; *n* = 27) and the bottom two quintiles (Low-BD-PRS; *n* = 27). To obtain the optimal methylation signature, PMPS were calculated utilising three *p* value thresholds from discovery EWAS summary statistics (*p*_T_ < 0.002, *p*_T_ < 0.01 and *p*_T_ < 0.05; representing *n* = 72, *n* = 389 and *n* = 1957 CpGs, respectively). Association with PMPS (dependent variable) was examined using univariate GLM, including BD-PRS strata as factor, with age, sex, tissue source, and blood-cell counts as covariates.

### Validation Set 2

To determine whether the PMPS indexed effects related to the development of psychopathology, 82 independent unrelated individuals of European ancestry were selected from batch 4. BD cases were recruited in Australia, and 68% of HR and controls were from US sites. A ‘BD-syndromic’ group (*n* = 44) comprised BD cases (*n* = 20) plus HR individuals who developed BD-related psychopathology (*n* = 24), and a ‘disorder-free’ group (*n* = 38) comprised controls (*n* = 26) plus HR who remained well (*n* = 12).

PMPS were generated using *p*_T_ < 0.05 (*n* = 1,891 CpGs, after QC exclusions), and compared between clinical groups: BD vs. CON, and extended ‘BD-syndromic’ vs. ‘disorder-free’ groups, using univariate GLM, as described above. BD-PRS and a BD-PRS×Group interaction term were also added to the model.

Sixty-six individuals in Validation Set 2 (80%) completed the FACES-II scale [53]. Exploratory regression analysis evaluating the impact of family environment, BD-PRS and psychopathology-group (and their interaction terms) on PMPS, employed a general linear model including age and sex as covariates.

## Results

### Polygenic risk scores

BD-PRS at *p*_T_ = 0.105 optimally distinguished the independent BD group (*n* = 264) from controls (*n* = 1115), with *p* = 5.36 × 10^−12^ and *R*^2^ = 0.057 (*n* = 214,928 SNPs; Fig. S1). The optimal MDD-PRS (for BD vs. controls) was at *p*_T_ = 0.017 (*n* = 187,029 SNPs, *p* = 1.89 × 10^−8^, *R*^2^ = 0.037), and the cross-disorder-PRS was optimal at *p*_T_ = 0.229 (*n* = 269,339 SNPs, *p* = 1.76 × 10^−10^, *R*^2^ = 0.049). At a group level, BD-PRS distinguished HR (*n* = 355) from both BD and control groups, where HR had higher mean BD-PRS than controls (*n* = 1699; GEE *p* = 0.0003) and lower BD-PRS than BD (*n* = 319; GEE *p* = 0.0006). The BD-PRS exhibited an overlapping population distribution across clinical groups (Fig. 2).

### Group comparisons: High vs. Low-BD-PRS strata within HR participants

Within the HR group, the High-BD-PRS stratum had significantly higher mean MDD-PRS (ANCOVA *F* = 5.32, *p* = 0.024, *η*^2^*p* = 0.064) and cross-disorder-PRS (ANCOVA *F* = 4.57, *p* = 0.035, *η*^2^*p* = 0.055) than the low-BD-PRS stratum. Epigenetically inferred smoking status analysis identified only two probable smokers, and these were equally represented in PRS group strata (Fig. S4). There was no difference in global functioning (GAF) between High-BD-PRS vs. Low-BD-PRS groups (*M* ± SD = 86.0 ± 9.3 vs. 84.8 ± 8.4, *p* = 0.54) (Fig. S6).

### Differentially methylated positions: high vs. low-BD-PRS strata within HR

**Fig. 3:**
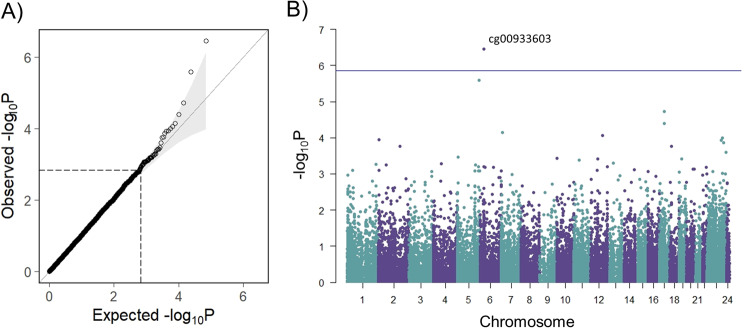
PRS-stratified EWAS in high-risk participants. **A** Quantile-Quantile plot, indicating observed vs. expected *p* values from 35,907 probes. Bias and inflation corrected to −0.00049 and 1, respectively. The dashed line indicates the inflection point at *p* < 0.002. **B** Manhattan plot indicating the genomic location of differentially methylated probes. The vertical line indicates the multiple-testing correction threshold at *α* = 0.05 for epigenome-wide association, based on 35,907 probes (*p* < 1.39 × 10^−6^). The location of the top DMP, cg00933603, is indicated. Covariates included age, sex, MDS C1 and C2, tissue source, six blood-cell components, and four surrogate variables. log logarithm; *P* *p* value.

EWAS was performed using a linear regression framework, using the final probe set of 35,907 variable CpGs that were blood-brain correlated. Following minor correction for bias and inflation (−0.0022 and 0.97, respectively; corrected to −0.00049 and 1, respectively), one probe (cg00933603, *p* = 3.54 × 10^−7^) exceeded the epigenome-wide association threshold (*p*_T_ < 1.39 × 10^−6^; FDR = 0.026) (Fig. 3). The quantile-quantile inflection point was defined at *p* < 0.002. Post-hoc adjustment for epigenetically-inferred smoking exposure (using β_cg05575921_ as a covariate) did not substantially alter the findings (cg00933603, *p* = 3.10 × 10^−7^; Table S2, Fig. S5).

### Putative biological impact of differentially methylated genes

The DMPs above the inflection point (*p* < 0.002; *n* = 82; Table S2) and nominally significant DMPs (*p* < 0.05; *n* = 1957) were physically and functionally mapped to 66 and 1260 genes, respectively. Genes with DMPs above the inflection included *MLC1, ESR1 KCKN5, L1CAM, CPEB1* and *GABBR2*, previously associated with BD [73] (Table S2; Fig. S7). EWAS-significant probe cg00933603 lies in exon 2 of *VARS2*, and has a *cis*-mQTL SNP rs2532928 (formerly known as rs116537083) [74], that correlates with *VARS2* cortical expression (GTEx v8; [75] *p* = 9.1 × 10^−29^; Fig. S8).

In FUMA analyses, the 1183 DMPs were enriched in genes previously associated with several relevant neurobehavioral phenotypes in the *GWAS catalog*, including schizophrenia, chronotype and general risk tolerance, which were represented in the top 15 enrichments (adjusted *p* < 0.01; Table 1 and Table S3). The genes that mapped to DMPs were mostly upregulated in brain regions, including the hypothalamus, anterior cingulate cortex and amygdala (adjusted *p* = 4.40 × 10^−16^, 7.08 × 10^−16^ and 1.69 × 10^−15^, respectively; Fig. S9). Top gene ontology enrichment categories from FUMA included synaptic and neuronal cellular components; canonical pathways involving synaptic function/interactions and signal transmission; and biological processes relating to cell-cell adhesion and signalling functions (Table 2, Table S4).

**Table 1:** Summary of neurobehavioral phenotypes within the top 50 ranked enrichments from FUMA *GWAS catalog*.

Gene set	*N* genes	*N* overlap	*P*	adj*P*	RANK
Primary EWAS^a^	1183		DMP *p* < 0.05		
Schizophrenia	827	52	6.63E−06	0.0012	10
Chronotype	556	39	8.22E−06	0.0013	11
General risk tolerance (MTAG)	248	22	2.52E−05	0.0030	15
Hippocampal sclerosis	9	4	1.26E−04	0.0094	24
Feeling nervous	40	7	2.84E−04	0.0191	27
Late-onset Alzheimer’s disease	53	8	3.06E−04	0.0198	28
Response to antidepressants (symptom improvement)	30	6	3.65E−04	0.0229	29
Depression (quantitative trait)	12	4	4.56E−04	0.0267	31
Cognitive ability, years of educational attainment or schizophrenia (pleiotropy)	197	16	8.08E−04	0.0396	37
Risk-taking tendency (4-domain PCA)	92	10	8.56E−04	0.0409	38
Feeling worry	48	7	8.95E−04	0.0417	39
Anger	24	5	9.37E−04	0.0425	40
Bipolar disorder	656	37	1.05E−03	0.0443	43
Psychosis (atypical)	15	4	1.16E−03	0.0459	45
Response to anti-depressant treatment in major depressive disorder	15	4	1.26E−04	0.0459	46
Supplementary EWAS^a^	1157		DMP *p* < 0.008		
General risk tolerance (MTAG)	248	26	1.66E−08	2.16E−06	13
Chronotype	556	42	1.78E−08	2.16E−06	15
Hippocampal atrophy	33	8	3.34E−06	2.19E−04	27
Morning person	202	19	6.83E−6	3.54E−04	35
Bipolar disorder	656	40	8.60E−06	4.34E−04	36
Major depressive disorder	210	19	1.19E−05	5.41E−04	40
Response to antidepressants (symptom improvement)	30	7	1.82E−05	7.15E−04	46
Late-onset Alzheimer’s disease	53	9	1.85E−05	7.15E−04	47

*N genes* number of genes in category, *N overlap* number of DMP genes in category, *P* *p* value, *adjP* adjusted *p* value, *DMP* differentially methylated probe, *RANK* rank of enrichment category on the basis of adjusted *p* value.

^a^Data are presented for an equivalent number of genes in both primary and supplementary EWAS that map to differentially methylated probes (DMPs) at specified thresholds. Full outputs of *GWAS catalog* enrichments are provided in Supplementary Tables S3 and S6.

**Table 2:** FUMA top 10 gene ontology (GO) and canonical pathway enrichment categories of ~1,183 genes that map to differentially methylated probes in primary EWAS (left) and comparable gene list from supplementary EWAS that included all CpGs regardless of blood-brain correlation (right).

RANK	Gene set	*N* overlap (*N* genes)	*p*	adj*P*	Gene Set	*N* overlap (*N* genes)	*p*	adj*P*
	Primary EWAS (blood-brain correlated CpGs)^a^				Supplementary EWAS (all CpGs)^a^			
GO biological processes								
1	Biological adhesion^b^	128 (1404)	7.88E−26	5.79E−22	Neurogenesis	152 (1594)	1.70E−32	1.25E−28
2	Homophilic cell adhesion via plasma membrane adhesion molecules	41 (165)	1.37E−24	5.04E−21	Neuron differentiation	128 (1343)	2.47E−27	9.08E−24
3	Cell–cell signalling	137 (1638)	4.77E−24	1.17E−20	Positive regulation of biosynthetic process	160 (1966)	1.78E−26	4.37E−23
4	Cell–cell adhesion via plasma membrane adhesion molecules	50 (271)	2.20E−23	4.05E−20	Positive regulation of gene expression	154 (1955)	4.99E−24	9.18E−21
5	Cell–cell adhesion	87 (819)	4.78E−22	7.03E−19	Central nervous system development	99 (972)	2.25E−23	3.31E−20
6	Synaptic signalling	73 (712)	8.10E−18	9.92E−15	Sensory organ development	70 (535)	7.72E−23	9.46E−20
7	Ion transport	121 (1663)	1.53E−16	1.61E−13	Positive regulation of RNA biosynthetic process	132 (1592)	1.43E−22	1.51E−19
8	Positive regulation of molecular function	121 (1740)	4.19E−15	3.85E−12	Animal organ morphogenesis	100 (1030)	4.63E−22	4.25E−19
9	Synapse assembly	31 (175)	1.63E−14	1.33E−11	Biological adhesion^b^	120 (1404)	1.24E−21	1.01E−18
10	Synapse organization	47 (404)	6.41E−14	4.71E−11	Growth	96 (979)	1.62E−21	1.19E−18
GO cellular components								
1	Intrinsic component of plasma membrane	145 (1697)	2.26E−26	2.26E−23	Neuron part^b^	150 (1709)	3.48E−28	3.48E−25
2	Synapse^b^	107 (1169)	8.30E−22	4.16E−19	Cell junction^b^	113 (1275)	1.23E−21	6.17E−19
3	Neuron part^b^	130 (1709)	2.66E−19	8.88E−17	Plasma membrane region^b^	104 (1185)	1.16E−19	3.14E−17
4	Cell junction^b^	106 (1275)	1.53E−18	3.83E−16	Synapse^b^	103 (1169)	1.33E−19	3.14E−17
5	Synapse part^b^	85 (932)	2.05E−17	4.11E−15	Neuron projection^b^	110 (1301)	1.57E−19	3.14E−17
6	Neuron projection^b^	102 (1301)	3.68E−16	6.15E−14	Cell projection part^b^	115 (1438)	1.56E−18	2.60E−16
7	Plasma membrane region^b^	94 (1185)	2.81E−15	4.01E−13	Intrinsic component of plasma membrane	124 (1697)	7.63E−17	1.09E−14
8	Postsynapse	61 (610)	1.35E−14	1.68E−12	Somatodendritic compartment	77 (818)	1.82E−16	2.27E−14
9	Cell projection part^b^	103 (1438)	8.84E−14	9.84E−12	Cytoskeletal part	119 (1639)	5.43E−16	6.04E−14
10	Whole membrane	111 (1647)	4.82E−13	4.82E−11	Synapse part^b^	81 (932)	2.69E−15	2.70E−13
GO molecular function								
1	Calcium ion binding	79 (693)	5.41E−22	8.91E−19	Sequence-specific DNA binding^b^	106 (1114)	1.15E−22	1.90E−19
2	Ribonucleotide binding^b^	123 (1885)	2.23E−13	1.83E−10	Regulatory region nucleic acid binding^b^	91 (934)	2.96E−20	2.43E−17
3	Drug binding^b^	112 (1718)	3.09E−12	1.70E−09	DNA binding transcription factor activity	128 (1691)	1.37E−18	7.49E−16
4	Adenyl nucleotide binding	101 (1536)	2.41E−11	9.91E-09	Sequence-specific double-stranded DNA binding^b^	83 (860)	2.59E−18	1.06E−15
5	Sequence-specific DNA binding^b^	78 (1114)	3.01E−10	9.28E−08	Double-stranded DNA binding^b^	88 (952)	3.60E−18	1.19E−15
6	Regulatory region nucleic acid binding^b^	69 (934)	3.54E−10	9.28E−08	Drug binding^b^	125 (1718)	7.84E−17	2.01E−14
7	Transmembrane transporter activity	74 (1038)	3.95E−10	9.28E−08	Ribonucleotide binding^b^	133 (1885)	8.55E−17	2.01E−14
8	Double-stranded DNA binding^b^	69 (952)	7.90E−10	1.62E−07	Adenyl nucleotide binding	114 (1536)	5.06E−16	1.04E−13
9	Sequence-specific double-stranded DNA binding^b^	64 (860)	1.17E−09	2.13E−07	Cytoskeletal protein binding	83 (948)	7.90E−16	1.44E−13
10	Ion transmembrane transporter activity	64 (870)	1.84E−09	2.76E−07	Identical protein binding	118 (1706)	2.24E−14	3.69E−12
Canonical pathways (Reactome)								
1	Neuronal system^b^	52 (410)	7.87E−17	1.73E−13	Neuronal system^b^	44 (410)	8.06E−12	1.77E−08
2	Protein protein^b^ interactions at synapses	21 (87)	5.20E−13	5.71E−10	Extracellular matrix organization	35 (299)	1.03E−10	1.13E−07
3	Transmission across chemical synapses^b^	33 (269)	8.46E−11	6.20E−08	Transport of small molecules	55 (728)	1.31E−08	9.61E−06
4	Neurotransmitter receptors and postsynaptic signal transmission^b^	28 (204)	1.66E−10	9.10E−08	Axon guidance	45 (551)	2.90E−08	1.38E−05
5	Neurexins and neuroligins	15 (56)	2.21E−10	9.71E−08	Developmental biology	72 (1100)	3.14E−08	1.38E−05
6	Activation of NMDA receptors and postsynaptic events	17 (92)	6.95E−09	2.55E−06	NABA matrisome^b^	68 (1024)	4.42E−08	1.62E−05
7	Disease	68 (1072)	1.88E−07	5.92E−05	Signalling by receptor tyrosine kinases	39 (468)	1.43E−07	4.17E−05
8	NABA core matrisome^b^	26 (274)	1.39E−06	0.000382	Protein protein interactions at synapses^b^	15 (87)	1.52E−07	4.17E−05
9	CREB1 phosphorylation through NMDAR-mediated activation of RAS signalling	8 (28)	2.21E−06	0.00054	Neurotransmitter receptors and postsynaptic signal transmission^b^	23 (204)	3.14E−07	7.29E−05
10	GPCR ligand binding	35 (453)	2.94E−06	0.000598	Transmission across chemical synapses^b^	27 (269)	3.32E−07	7.29E−05

*RANK* rank of enrichment category on the basis of adjusted *p*-value, *N genes* number of genes in category, *N overlap* number of DMP genes in category. *DMP* differentially methylated position, *P* *p* value, *adjP* adjusted *p* value.

^a^Data are presented for an equivalent number of genes in both primary and supplementary EWAS that map to differentially methylated probes. Full outputs of GO and canonical pathway enrichment are provided in Tables S4 and S7.

^b^Categories that are represented in top 10 for both primary EWAS using blood-brain selected probes, and unselected supplementary EWAS.

### Supplementary EWAS: post-hoc sensitivity analysis for interpretation of DMP functional enrichments

No DMP survived multiple-testing correction in the supplementary EWAS using 214,352 probes regardless of blood-brain correlation (Fig. S10). The top DMP from the primary PRS-EWAS (cg00933603; *p* = 3.54 × 10^−7^) was the third top DMP (*p* = 4.38 × 10^−6^) in this secondary EWAS (Table S5).

Pathway analysis in FUMA showed that brain tissues continue to exhibit the strongest enrichment with an equivalently-sized gene list derived from DMPs regardless of blood–brain correlation. The top brain regions were cortex, anterior cingulate cortex and amygdala (*p* = 1.58 × 10^−14^, *p* = 1.35 × 10^−11^ and *p* = 2.39 × 10^−11^, respectively; Fig. S11). Gene ontology enrichments implicate neuronal-related functions and *GWAS catalog* overlaps, with chronotype represented in the top 15 enrichments (Tables 1 and 2, Tables S6 and S7).

### Test of generalisability of epigenetic signature using poly-methylomic profile score

#### Validation Set 1

PMPS calculated using three *p* value thresholds (*p*_T_) were normally distributed (Shapiro–Wilk *p* > 0.05). At the most stringent threshold (*p*_T_ = 0.002), no association between PMPS and BD-PRS strata in controls was observed (*F* = 1.00, *p* = 0.323, *η*^*2*^*p* = 0.023). However, the PMPS at *p*_T_ = 0.05 (*n* = 1,891 CpGs) showed stronger associations with BD-PRS strata (*F* = 11.57*, p* < 0.002*, η*^*2*^*p* = 0.212) than the PMPs at *p*_T_ = 0.01 (*F* = 7.11*, p* = 0.011*, η*^*2*^*p* = 0.142); PMPS was higher in controls with High-BD-PRS (*p*_T_ = 0.05; *M* ± SE = 11.84 ± 1.27) compared to low BD-PRS (*M* ± SE = 8.17 ± 1.34). Thus we considered *p*_T_ = 0.05 to be optimal for use in Validation Set 2 (Table S9).

#### Validation Set 2

##### Associations between PMPS and PRS or clinical status in BD and CON groups

PMPS at *p*_T_ = 0.05 was normally distributed within each clinical group (Shapiro-Wilk *p* > 0.05). PMPS did not differ between BD and control groups in a basic model that did not covary for BD-PRS (*F* = 2.39*, p* = 0.131*, η*^*2*^*p* = 0.066; Table S9). Following the inclusion of BD-PRS and BD-PRSxGroup term in the model, a significant association between PMPS and BD group was observed (*F* = 7.20*, p* = 0.011*, η*^*2*^*p* = 0.184; Table S9), in conjunction with a significant BD-PRSxGroup interaction (*F* = 7.07*, p* = 0.012*, η*^*2*^*p* = 0.181) and a suggestive effect of BD-PRS (*F* = 3.99*, p* = 0.054*, η*^*2*^*p* = 0.111).

##### Associations between PMPS and PRS or clinical status in ‘BD-syndromic’ and ‘disorder-free’ groups

Extended-clinical group definitions were utilised based on BD-psychopathology in HR (Table S8). HR meeting threshold or sub-threshold diagnoses (HR_unwell_) demonstrated lower functional capacity (CGI scores) than HR who remained well (HR_well_) (Fig. S12). Overall, the ‘BD-syndromic’ group had poorer global functioning (GAF) than the ‘disorder-free’ group (*M* ± SD = 74.0 ± 14.7 vs. 92.7 ± 6.0, *p* = 2.75 × 10^−6^) (Fig. S6).

PMPS was not associated with psychopathology-group without BD-PRS in the model (*F* = 0.39*, p* = 0.533*, η*^*2*^*p* = 0.006). Following the inclusion of BD-PRS and BD-PRS × Group term in the model, a significant association between PMPS and BD group was observed (*F* = 5.55*, p* = 0.021*, η*^*2*^*p* = 0.076; Table S9), in conjunction with a significant BD-PRS × Group interaction (*F* = 5.52*, p* = 0.022*, η*^*2*^*p* = 0.075) and a non-significant effect of BD-PRS (*F* = 2.33*, p* = 0.132*, η*^*2*^*p* = 0.033).

A post-hoc sensitivity analysis of the impact of psychiatric medication use on PMPS was conducted within the BD-syndromic group, which showed no evidence of medication effects on PMPS (Supplementary Results). Exploratory regression analysis in the subset of participants with FACES data (*n* = 66; omnibus model *p* = 0.013), showed that PMPS was not associated with FACES (Wald-*χ*^2^ = 0.671, *p* = 0.413), but BD-PRS, psychopathology-group, and BD-PRS × Group were significant predictors of PMPS (Wald-χ^2^ = 9.55, *p* = 0.002; Wald-χ^2^ = 9.29, *p* = 0.002; Wald-χ^2^ = 10.17, *p* = 0.001; respectively), while the FACES × Group interaction was non-significant (Wald-χ^2^ = 3.24, *p* = 0.072).

### Sensitivity analysis for population stratification

To ensure our results were robust to residual population stratification within our selected study samples, the most divergent four ‘nearest neighbour’ individuals were identified by pairwise IBS distances, and excluded if Z > 4 (*n* = 5 individuals total; *n* = 1 discovery EWAS, *n* = 3 validation set 1, *n* = 1 validation set 2). Removal of one participant from discovery EWAS with outlier values on C4 did not substantially alter the findings (effect size r = 0.9999; absolute β difference ~1.43 ± 1.49%). For validation set 1, three ‘nearest neighbour’ exclusions resulted in a slight attenuation of association between PMPS at *p*_T_ = 0.05 and BD-PRS strata (*F* = 8.946*, p* = 0.005*, η*^*2*^*p* = 0.183). For validation set 2, ‘nearest neighbour’ exclusions (*n* = 1) revealed almost identical results and effect sizes (*η*^*2*^*p* ± 0.001).

## Discussion

After stratifying HR individuals based on their personal burden of BD-associated common genetic variants indexed by BD-PRS and restricting the epigenome search space to variable probes with blood-brain correlation, we found a single epigenome-wide significant differentially methylated probe (cg00933603), located in an active regulatory element in exon 2 of the *VARS2* gene, which lies in the major histocompatibility complex region (hg19/chr6:25–34 Mb). *VARS2* encodes a mitochondrial aminoacyl-tRNA synthetase involved in mitochondrial protein synthesis. Mitochondrial abnormalities are evident in BD [76] and other psychiatric illnesses [77], and loss-of-function mutations in *VARS2* have been previously associated with mitochondrial encephalopathies [78], epilepsy [79], and schizophrenia [80]. Furthermore, an EWAS of depressive symptoms in 724 monozygotic Danish twins identified a differentially methylated region in a putative active enhancer of *VARS2*, which spanned 9-probes including cg00933603 [81]. In our study, *VARS2* was hypomethylated in individuals with a high polygenic burden for BD. Hypomethylation in the 5′ region of a gene can promote gene expression [82], which in the case of *VARS2*, might play a role in phasic dysregulation of mitochondrial bioenergetics associated with BD [83]. Further characterisation of this ubiquitously expressed functionally relevant gene in the pathophysiology of BD is required.

Beyond the top DMP, disease-associated genetic variants may broadly impact epigenetic processes, supporting the supposition that DNA methylation may mediate genetic risk [84], potentially via long-range epigenetic networks [85]. Of 1,183 genes that mapped to nominally significant DMPs, some were previously associated with BD or schizophrenia, including *MLC1*, *ESR1*, *KCKN5, L1CAM, CPEB1* and *GABBR2* [73]. In addition, 693 of these genes contained CpGs independently nominally associated with later development of BD and MDD in individuals at high familial risk of mood disorder [43]. Although overlap between the specific DMPs implicated in that prior study and our present analysis was low, gene-level convergence provides supporting evidence for specific differentially methylated genes in developing affective psychopathology.

While the subgrouping of HR participants was primarily focused on genetic predisposition to BD, the BD-PRS strata did not exclusively index BD-associated risk—a higher burden of cross-disorder and MDD-associated variants was observed—which supports a relative lack of specificity of PRS. It is therefore possible that the methylation signatures identified herein may show pleiotropic effects across these related psychiatric disorders.

Functional analyses indicated that genes differentially expressed in the brain were over-represented amongst the differentially methylated genes, particularly in the hypothalamus and anterior cingulate cortex (ACC). The hypothalamus is part of the hypothalamic-pituitary-adrenal (HPA) axis, the key stress-response system, which has been shown to be dysregulated in mood disorders [86]. In addition, the ACC plays an important role in cognitive functions and emotional regulation. Structural imaging meta-analyses have shown grey matter reduction in ACC [87], and smaller hippocampal volumes associated with BD [88, 89], thus methylation changes impacting these tissues are potentially of relevance. Overlap between genes mapped to DMPs and genes implicated in relevant phenotypes via GWAS—including BD and schizophrenia, chronotype, and risk tolerance—suggest convergence of epigenetic and genetic signals. Sleep disruption and chronotype have long been posited as hallmark features of BD [90], as well as targets of psychiatric medication [91], and may influence medication response [92]. Risky behaviour is a common symptom in manic phases and is genetically correlated with BD [93], highlighting relevant pathways within our defined epigenetic signals.

Restricting the discovery EWAS to putatively functionally relevant CpGs that reflect methylation status in the primary disease-affected tissue [62, 63] enabled reduction of the EWAS search space and application of a more permissive epigenome-wide significance threshold than previously modelled [71], but may consequentially bias enrichment analyses. Our secondary EWAS employed *all* variable CpGs, regardless of blood-brain correlation, and revealed similar over-representation in brain tissue, pathway enrichments, and overlap of top DMPs with an equivalent-sized gene list; supporting the primary methodological approach.

Utilising nominally differentially methylated CpGs from PRS-stratified EWAS in generating a poly-methylomic profile score (PMPS) [72]—akin to calculating PRS from GWAS summary statistics for *genotypic* data [14]—we validated the impact of BD-PRS on methylation signature. We found significant associations between BD-PRS and PMPS in controls, demonstrating the generalisability of the PMPS as an index of PRS, regardless of family history of BD. Consistent with the dependency of the PMPS on PRS background, when BD-PRS was included in the statistical models testing differences between groups in PMPS, a higher mean poly-methylomic profile score was observed in those with BD-symptomatology compared to disorder-free individuals. This highlights the important interplay between environmental, epigenetic and genetic risk factors, which may lead to the development of psychopathology, and is consistent with a multifactorial liability model [94].

While there was no significant effect of FACES on PMPS in our exploratory analysis, this study cannot rule out the potential influence of family environment on methylation signature, given the small sample size with available FACES that was used. The PMPS appears to at least partially reflect environmental differences related to the burden of BD-associated variants, however, increasing the sample size for discovery EWAS may further elucidate the mechanisms underlying the development of psychopathology. Longitudinal investigation of epigenetic markers in HR individuals based on their clinical status over time may also reveal useful epigenetic signatures of the BD prodrome.

### Limitations

This study was restricted to participants of European-ancestry, due to substantial ethnicity-specific effects on PRS [60]. We note that residual population substructure not indexed by the covariates we employed (i.e. the first two MDS components or four surrogate variables of methylation data) may influence the results reported herein. However, expanding the existing 15 essential covariates to include additional MDS components would further reduce the ‘subjects per variable’ ratio, and compromise model stability and accuracy of estimation of regression coefficients [95]. This trade-off in design of our statistical model for analysis of this cohort may lead to under-correction of fine-scale population structure that is evident in European population [96, 97], although sensitivity analysis showed little effect of excluding potential outliers. To permit the study design to include both discovery and validation samples, sub-setting of the cohort was required, which limited sample size. While we employed a Bayesian method to reduce potential for EWAS bias [67], the methylation effects reported herein require validation. We mapped DMPs both physically and functionally [68], to comprehensively define genes associated with DMPs in both coding and non-coding genomic regions. However, genes were not prioritised for inclusion in enrichment analysis based on the number nor location of DMPs within each mapped gene, nor was the list restricted to one gene per DMP, thus enrichment signals may have been impacted by inclusion of genes whose expression was not affected by DMPs. Furthermore, in silico mapping of potentially cross-reactive CpG probes to homologous genome locations [98] may impact gene annotations and enrichments. Moreover, FUMA does not account for the number of CpGs per gene, therefore enrichment results may be biased towards categories with larger genes that contain more probes (i.e., with greater stochastic chance to be differentially methylated) [99].

One universal limitation of epigenome-wide studies in psychiatric disorders is the accessibility to brain tissue, as there is considerable variation in DNA methylation across tissues and cell types [100, 101]. We addressed this design limitation by covarying for inter-individual variability in blood-cell components, and focusing on CpGs which are blood-brain correlated [62]. However, use of blood-derived DNA may exclude relevant brain-specific CpGs that are not variable in this surrogate tissue. We acknowledge that: (1) participants were at different follow-up points, with potential limitations on capacity for defining ‘syndromic’ status that is dependent on length of clinical follow-up, and (2) PMPS reflects *baseline* methylation signatures, and timing of emergence of symptoms in relation to proximity to blood draw is not accounted for. Furthermore, while only two participants in the discovery EWAS were epigenetically inferred to be probable smokers (and were equally represented in PRS strata), smoking exposure may be a relevant confounder for future replication. Finally, psychiatric medication use was not controlled for due to the small numbers of individuals exposed, and although sensitivity analysis indicated non-significant effect of medication on PMPS, this remains an important caveat.

Although discovery EWAS in HR participants stratified by BD-PRS can reveal epigenetic markers associated with genetic risk for developing BD, many genetic variants that may impact BD risk are not indexed by PRS [18]. Indeed, familial risk encompasses all classes of genomic variation—including structural and rare variants—which may track more closely than PRS with disease-status in families with strong family history [21]. While we excluded annotated ‘probe SNPs’ from our analysis [102], the existence of polymorphic genotypes (e.g., rare or recently discovered DNA variants) that are present in our cohort and underlie CpGs could potentially lead to bias [103]. Conversely, we note that standard clumping procedures employed herein (based on *r*^2^) for generation of PRS included multiple MHC variants, thus PRS group differences may be inflated by multiple genetic effects from this region, which may amplify apparent effect sizes of methylation differences in the MHC region. The MHC region was also not excluded in FUMA enrichment analysis. Finally, limited availability of family environment data impacted power to detect effects, which require larger samples to definitively characterise. Thus extension to larger cohorts, and potential examination of the impact of other environmental factors, including stressful life-events, should be considered in future.

## Conclusions

To our knowledge, this is the first report to investigate DNA methylation differences amongst individuals at high risk of BD, in the context of their personal polygenic background for BD. While only a single site in the *VARS2* gene exceeded epigenome-wide significance, many CpG sites were nominally differentially methylated, which related to neurological pathways and functions associated with risk of psychopathology. Methylation profiling in independent validation sets confirmed the relationships between methylation signatures and genetic background, finding that methylation profiles may also partially reflect differences in family environment. Further larger-scale studies are needed to examine the impact of environmental factors in the relationship between familial risk and development of psychopathology.

## Supplementary information

Supplementary material

Supplementary Tables

## Data Availability

The data that support the findings of this study are available in the UNSW data-archive “ResData” at www.dataarchive.unsw.edu.au, under Research Data Management Plan reference number D0237303, or are available on request from the authors.
