# Supplementary material for: Epigenetic signatures relating to disease-associated genotypic burden in familial risk of bipolar disorder
Source: Transl Psychiatry. 2022 Aug 3;12:310. doi: 10.1038/s41398-022-02079-6 (PMC9349272; doi:10.1038/s41398-022-02079-6)
Supplement: Supplementary file 1 — Supplementary material [file 41398_2022_2079_MOESM1_ESM.docx]

**Supplementary Material**

“Epigenetic signatures relating to disease-associated genotypic burden in familial risk of bipolar disorder”

Sonia Hesam-Shariati, Bronwyn J. Overs, Gloria Roberts, Claudio Toma, Oliver J. Watkeys, Melissa J. Green, Kerrie D. Pierce, Howard J. Edenberg, Holly C. Wilcox, Emma K. Stapp, Melvin G. McInnis, Leslie A. Hulvershorn, John I. Nurnberger, Peter R. Schofield, Philip B. Mitchell, Janice M. Fullerton*.

*** Corresponding Author:** Janice M. Fullerton, Neuroscience Research Australia, Margarete Ainsworth Building, Barker Street, Randwick, Sydney, NSW 2031, Australia; E j.fullerton(at)neura.edu.au; T +61 (0)2 9399 1836; F +61 (0)2 9399 1005

**Supplementary Methods**

**DNA extraction**

Peripheral blood samples were collected in EDTA tubes for processing and DNA extraction. Samples were processed either as: 1) whole blood, or 2) separated to isolate lymphocytes for EBV transformation, where a cell line and DNA was required from a single collection tube. Where a cell line was made, the blood was first diluted with saline, then centrifuged through a ficoll layer to isolate the lymphocyte layer for Epstein-Barr virus (EBV) transformation (comprising predominantly granulocytes; Figure S3). A layer of lymphocytes left on top of the red blood cells and all of the leftover ficoll layer cells (i.e. red and white cells from the ficoll/PBS/plasma layer, including pelleted cells and depleted of granulocytes; Figure S3) was either processed for DNA extraction or frozen (in case of cell line failure or used as backup if two EDTA tubes were sent and the DNA extraction from the first whole-blood tube failed). In some instances, DNA was extracted from the isolated lymphocyte population after ficoll separation, but before EBV transformation. DNA was extracted by Genetic Repositories Australia, using either the Qiagen Autopure® LS or a Puregene salting out methodology (Qiagen, Chadstone, Victoria, Australia), according to manufacturers’ instructions. While cell composition has a major impact on methylation variability, DNA extraction procedures and sample storage have been shown to have minimal impact on variability.[1]

For US participants, samples were processed for DNA extraction either as: 1) whole blood, 2) saliva, or 3) EBV-transformed cell lines from cryopreserved lymphocytes, by the Rutgers University Cell and DNA Repository (RUCDR Infinite Biologics, New Brunswick, NJ, USA; now operating as Infinity BiologiX or IBX) using standard automated procedures [requestID=2689; details of services are available at [https://www.rucdr.org/](https://www.rucdr.org/biosample-processing/nucleic-acid-extraction-and-analysis) or <https://ibx.bio/>). Samples derived from saliva were excluded from selection for methylation array due to their ectodermal origin, instead focusing on samples from blood-derived sources of mesodermal origin for Validation Set 2.

**SNP Genotyping and imputation**

Genotyping was performed as previously described.[2] Briefly, all genotyping employed the Illumina PsychArray-24 BeadChip. Genotype calling and quality control employed the Psychiatric Genomics Consortium (PGC) ricopili pipeline (v8.0). Genotypes underwent filtering and quality control in PLINK (v1.90)[3] using ENIGMA2 protocols (<http://enigma.ini.usc.edu/>), and cohorts were combined before imputation. Pairwise identity-by-descent (IBD) analysis was employed to identify spurious participant relationships (pi_hat>0.1).

Imputation employed the Michigan Imputation Server (v1.0.3),[4] with 1000Genomes reference panel (Phase 3, v5), and phasing by Eagle v2.3. Additional post-imputation filtering removed SNPs that violated Hardy-Weinberg Equilibrium (*p*<0.000001), Minor Allele Frequency (MAF)<0.01 or imputation quality score (INFO)<0.8.

**Independent cohorts (used for generation of optimal PRS)**

For generation of PRS and identification of the optimal *p*-value threshold for single nucleotide polymorphisms (SNP) inclusion, a larger sample of independent BD case and control subjects genotyped on PsychArray-24 were employed. This collection included European-ancestry participants with established BD (*n*=264), as described in [5] as “neuc1”. BD cases were recruited at the Mood Disorder Unit, Prince of Wales Hospital in Sydney and the South Eastern Sydney-Illawarra Area Health Service (SESIAHS).[6-8] All cases received a lifetime diagnosis of BD according to the DSM-IV[9] criteria, on the basis of structured diagnostic interviews using the DIGS,[10] FIGS,[11] and the SCID,[12] and a consensus best-estimate diagnostic procedure. Healthy European-ancestry controls (*n*=1115) were recruited from the community and Twins Research Australia (TRA; TWIN-E study [13, 14]). Community controls had no personal lifetime history of a DSM-IV Axis-I diagnosis as determined by psychiatric interview, and no history of psychotic disorders among first-degree biological relatives. Controls from the TWIN-E study (*n*=751 unrelated) provided saliva samples for DNA and questionnaire data, but did not undergo formal psychiatric assessment and were not screened for a family history of mental illness. DNA was extracted by Genetic Repositories Australia using the Qiagen Autopure® LS, as described above.

**Multidimensional scaling and** **population stratification**

Multidimensional scaling analysis was performed in PLINK (v1.90)[3] to derive ethnicity components for the full genetic sample (n=2,800) as follows: 1) autosomal SNPs that passed quality control and excluded mhc region were pruned with *--indep-pairwise* within a 100-variant window, using a 25-variant shift, and a pairwise r^2^ threshold of 0.2, to identify independent SNPs, 2) MDS components were extracted employing the singular value decomposition (SVD) method using the *--MDS-plot* and *--cluster* options, taking into account IBS/IBD within the sample. MDS C1 and C2 were employed in downstream analysis as these were above the scree plot inflection point, and accounted for the majority of variance in SNP variability due to ethnicity, with eigenvalues accounting for 31.24 and 15.34% of variance respectively; C3-C20 had substantially lower eigenvalues, accounting for between 4.20-2.27% variance. Genotype files were merged with HM3_b37 reference files and MDS rerun to visualise clusters relative to HAPMAP population groups (Figure S2).

All individuals selected for EWAS discovery (n=82) were within ±6SD of the population mean of HAPMAP CEU individuals across MDS components 1-20 (with the exception of one C4 value), consistent with the threshold recommended to define outliers.[15] Furthermore, pairwise identity-by-state (IBS) distances amongst participants’ samples selected for discovery or validation sets were calculated from all autosomal SNPs that remained after pruning using the *--neighbour* option. Four nearest neighbours were identified for each individual based upon the pairwise IBS distance, with IBS distance values transformed into a Z score. No individual had a Z score greater than 6 amongst the four nearest neighbours. Individuals with a Z score greater than 4 were identified for sensitivity analysis (n=5 individuals total; n=1 discovery EWAS, n=3 validation set 1, n=1 validation set 2).

**Epigenome-wide methylation profiling and initial quality control**

Following bisulfite conversion of DNA from each individual, using the EZ-96-DNA methylation kit (Zymo Research, Orange, CA, USA), genome-wide DNA methylation was assessed using Epoch Microplate Spectrophotometer (Biotek, Winooski, VT). Genome studio methylation module software (Illumina, San Diego, CA, USA) was then used on all chips to extract signal intensities of probes, quality control and methylation measures.

The QC procedure involved excluding individual subjects for whom: 1) the predicted median methylation signal was in excess of three standard deviations from the regression line generated from methylated vs. unmethylated signal intensities (batch 1: *n*=1 HR male; batch 2-4: *n*=0), 2) more than 10% of probes had less than three beads (*n*=0 samples), 3) methylation signal intensities deviated from mean values for control probes (batch 1: *n*=1 HR female; batch 4: *n*=1 HR female; batch 2-3: *n*=0), 4) more than 10% of probes had a detection *p*-value > 0.01 (*n*=0 samples), 5) a sex mismatch or XY outlier status was detected (*n*=0 samples), 6) a genotype mismatch with genotypes derived from Illumina PsychArray (*n*=65 SNPs) was detected (batch 1-3: *n*=0; batch 4: *n*=6 samples, due to missing genotype data), or 7) were determined to be outliers after principal component analysis of methylation data within ShinyMethyl. Individual probes were excluded if: 1) >10% of samples showed detection *p*-values > 0.01 for that probe (*n*=1,259 *n*=295, *n*=441 and *n*=340 in batches 1, 2, 3 and 4 respectively), 2) >10% of samples demonstrated less than 3 beads at a given probe (*n*=374, *n*=28, *n*=136 and *n*=623 probes, in batches 1, 2, 3, and 4 respectively), 3) the probe was represented on only one of the two chip types employed (*n*=425,453). Finally, probes which were annotated in the Illumina manifest file (HumanMethylation450_15017482_v1-2) as ‘probe SNPs’ were excluded (*n*=6,456).

Of the 96 DNA samples assayed in Round 1, 4 duplicate samples were excluded, and a further 4 failed QC and were excluded (2 outliers on PCA of methylation data, and 2 due to QC failures), leaving 88 samples for analysis. All samples in Round 2 passed QC and were retained. Of the 127 DNA samples assayed in Round 3, one sentrix chip (*n*=8 samples) was an outlier on PCA of methylation data and was discarded, and 118 were retained. Of the 96 DNA samples assayed in Round 4, 7 failed QC (1 control probe deviation, 1 >3SD of mean Methylated/Unmethylated intensities, 5 had missing genotype data), and 7 were replicate HR samples selected from Rounds 1-3 and were excluded, leaving 82 samples for analysis. In total, 384 of the 415 samples were retained, of which 367 had genotype data and 306 were European-ancestry (*n*=180 HR, of whom 103 were singletons; *n*=126 controls, of whom 67 were singletons).

**Tissue source**

Of the 304 samples that passed quality control in rounds 1-3 (average age=20.49±5.21; 43% male; 175 HR, 129 CON), 20% had DNA derived from whole blood, 51% firstly underwent ficoll density gradient separation with DNA extracted from the cells in the ficoll/PBS/plasma “buffy coat” layer, and 28% were derived from the isolated lymphoblast cell population after ficoll separation. The breakdown of tissue source of samples included in discovery EWAS is presented in Table S1. The estimated cell component proportions from the discovery sample are presented in Figure S3.

For round 4 (average age=19.57±5.28 years; 48% male; 50 HR, 26 CON, 20 BD), 30% of the samples were derived from whole blood, 26% were ficoll separated, and 43% were derived from isolated lymphoblast cells.

For Validation Set 1 (controls of European-ancestry and unrelated), there were only 5 DNA samples that were derived from whole blood across rounds 1-3; thus these samples were excluded, and two tissue sources (ficoll- and lymphocyte-derived) were employed.

**Disease-gene association analysis**

Genes mapped to DMPs were cross-referenced against reports of disease-gene associations as catalogued in DisGeNET v7.0,[16] which includes data from Psychiatric disorders Gene-association Network (PsyGeNET),[17] [NHGRI-EBI GWAS Catalog](https://www.ebi.ac.uk/gwas/)[18] and GWAS database.[19] Diseases examined were bipolar disorder (diseaseID:C0005586) and schizophrenia (diseaseID:C0036341), yielding gene sets of 1,184 and 2,873 genes respectively.

The DMP genes were cross-matched with genes previously associated with BD and SCZ (DisGeNET v7.0), finding 77 genes that were previously associated with BD and 182 genes with schizophrenia, of which 59 were common between schizophrenia and BD (Figure S7).

**Family environment measures**

The Family Adaptability and Cohesion Evaluation Scales (FACES-II)[20] was employed at baseline to measure the emotional bonding between family members (cohesion) and their ability to change roles and structure during difficult times (adaptability). FACES-II is a 30-item self-report questionnaire, on a 5-point Likert-scale, which measures two dimensions of family behavior, cohesion and adaptability. *Cohesion* is defined as the extent to which family members are separated from or connected to the family, or the degree of emotional bonding that family members have toward one another. *Adaptability* is defined as the extent to which the family system is flexible and able to change its power structure, role relationships, and relationship rules in response to situational and developmental stress.

Scoring employed the FACES-II update which incorporates linear scoring norms and interpretation,[20] due to its higher alpha reliability and concurrent validity. A total score for the 14-item adaptability subscale (step 1: sum items 24 and 28; step 2: step 1 value from 12; step 3: sum all remaining even items except item-30; step 4: sum step 2 + step 3) places the respondent's family into one of eight sub-levels, which correspond to four levels with established cut-off points for adaptability (very flexible, flexible, structured, rigid). Likewise, a total score for the 16-item cohesion subscale (step 1: sum items 3, 9, 15, 19, 25 and 29; step 2: subtract 36 from step 1 value; step 3: sum all remaining odd items plus item-30; step 4: sum step 2 + step 3) places the respondent's family into one of eight sub-levels, which correspond to four levels of cohesion (very connected, connected, separated, disengaged). The FACES total score was computed from the sum of adaptability and cohesion subscales and used as a quantitative measure of family environment.

All participants (12-30 years old) were eligible to complete the FACES-II and a subset completed this scale at their baseline interview. Participants who only partially completed the scale were excluded from analysis.

**Supplementary Results**

**Supplementary EWAS: post-hoc sensitivity analysis for interpretation of DMP functional enrichments**

As the discovery EWAS employed a restricted set of 35,907 CpG probes that were blood-brain correlated, we reasoned that probe pre-selection might lead to skewing of functional enrichments in genes mapped to DMPs (particularly towards brain-related enrichments). Therefore, EWAS was repeated using 214,352 probes that passed QC (without applying the blood-brain correlation filter) employing identical procedures as the primary EWAS. The epigenome-wide multiple testing correction threshold was adjusted to *p*<2.33×10^-7^ for 214,352 tests at α=0.05.[21]

Following minor correction of bias and inflation (estimated bias=0.00097, inflation=1), no DMP survived multiple testing correction (Figure S10). However, the top DMP from the primary PRS-EWAS (cg00933603; *p*=3.54×10^-7^) was the third top DMP (*p*=4.38×10^-6^) in this secondary EWAS (Table S5).

Pathway analysis was performed employing a gene list of equivalent size to the primary EWAS (*n*=1,126) in order to infer the effect of probe pre-selection on functional enrichments (Tables 1-2, Tables S6-7). To create an equivalent-sized gene list, DMPs with *p*<0.0064 were physically and functionally mapped to 1,157 genes. Pathway analysis in FUMA showed that brain tissues continue to exhibit the strongest enrichment. However, in contrast to the primary EWAS enrichment which showed primarily up-regulation of brain expression, we observed significant enrichment in both up- and down-regulated genes in brain (Figure S11). The top brain regions included the cortex, anterior cingulate cortex and amygdala (*p*=1.58×10^-14^, *p*=1.35×10^-11^ and *p*=2.39×10^-11^,respectively)(Figure S11).

**Sensitivity analysis of psychiatric medication use on PMPS**

As control and HR participants were largely not exposed to treatment with psychiatric medications, analysis of the impact of medication effects was only informative within Validation Set 2. Post-hoc analysis of psychiatric medication use on PMPS was conducted within the BD-syndromic group, of whom 65% (*n*=29) had received any psychiatric medication (*n*=18 BD cases, *n*=11 HR; Table S8). The basic model employed PMPS as the outcome variable, and included sex, tissue, age, six cell counts as covariates (± BD-PRS, ± medication). There was no evidence of association between PMPS and any medication use in a model without BD-PRS (*F=*1.001*, p=*0.325*, η^2^p=*0.030). The model that included BD-PRS (without medication) showed a trend for BD-PRS on PMPS (*F=*3.728*, p=*0.062*, η^2^p=*0.104). The model that included both BD-PRS and medication revealed no evidence of medication effect on PMPS (*F=*0.186*, p=*0.669*, η^2^p=*0.006) but a small attenuation of BD-PRS effect on PMPS (*F=*2.763*, p=*0.107*, η^2^p=*0.082).

**Epigenetically-inferred current smoking index**

Smoking status was epigenetically-inferred using normalised β-values at cg05575921,[22] noting that *current* smoking status was not available in sufficient numbers of study participants to enable robust estimates of predictive accuracy in the present sample (n=36 out of 218 individuals; n=5 current users, all in Validation Set 2). Using published cutoffs of β<0.75 for *current* probable-smokers, as defined by Dawes *et al*.,[22] 2 probable-smokers were identified in the EWAS discovery sample of HR participants (2.5%; *n*=1 in each of the high and low BD-PRS groups respectively; and *n*=9 (9.7%) ambiguous), 0 probable-smokers in Validation Set 1 (control participants with β<0.75; *n*=9 (17%) were ambiguous) and 12 probable-smokers in Validation Set 2 (*n*=4 BD, *n*=7 HR, *n*=1 CON; *n*=25 were ambiguous)(Figure S4). Mean beta values for BD cases (β=0.798±0.107) were significantly higher than both control (β=0.883±0.045; *p*=0.002) and HR groups (β=0.883±0.062; *p*=0.002), but HR were no different to control (*p*=0.970).

While the predictive validity of this smoking index was not able to be empirically determined in this young cohort (aged 12-30), inclusion of the β_cg05575921_ as a covariate to account for epigenetically-inferred smoking exposure on PMPS in Validation Sets 1 and 2 yielded very similar results with regard to BD-PRS, group and BD-PRSxGroup effects, with slightly larger effect sizes (data not shown).

**Supplementary Tables**

See accompanying excel file for Supplementary Tables S1-S9.

Supplementary Table 1. Demographics of European-ancestry participants and tissue source in each analysis subset.

Supplementary Table 2. Top differentially methylated positions (p<0.002; above the inflection point) in High vs. Low BD-PRS group from primary EWAS.

Supplementary Table 3. Full output of FUMA enrichment of genes from GWAS catalog for DMP genes from primary EWAS of High vs Low BD-PRS groups in HR with restricted blood-brain correlated probe set.

Supplementary Table 4. Full output of FUMA enrichment of gene ontology (GO) and canonical pathway enrichment categories from primary EWAS of High vs Low BD-PRS groups in HR with restricted blood-brain correlated probe set.

Supplementary Table 5. Nominally significant differentially methylated positions (p<0.001) from the supplementary EWAS of 214,352 probes.

Supplementary Table 6. Full output of FUMA enrichment of genes from GWAS catalog for DMP genes from supplementary EWAS of High vs Low BD-PRS groups in HR with unrestricted blood-brain correlated probe set.

Supplementary Table 7. Full output of FUMA enrichment of gene ontology (GO) and canonical pathway enrichment categories from supplementary EWAS of High vs Low BD-PRS groups in HR with unrestricted blood-brain correlated probe set.

Supplementary Table S8. BD-related clinical diagnoses in each analysis subset, incorporating diagnosis timing at baseline and after annual follow-up clinical assessments.

Supplementary Table 9. Epigenetic signature using poly-methylomic profile score and associations between group, PRS and PRS×Group interactions.

**Supplementary Figures**


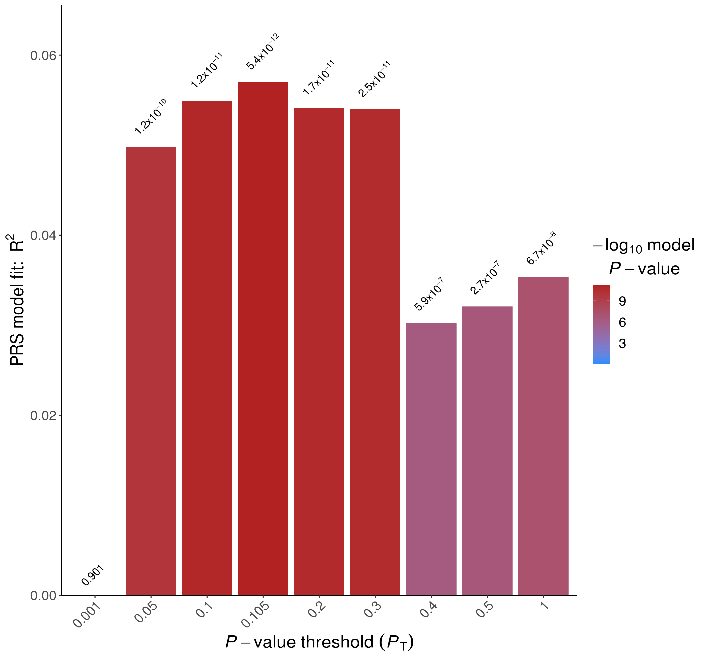


*Figure S1.* Bar plot from PRSice showing optimal p-value threshold for BD-PRS. Y-axis shows Nagelkerke pseudo R^2^ at various *p*-value thresholds, to determine the variance accounted for by the PRS. The PRSicev2 default interval step size of 0.00005 was reduced to 0.001 for optimal *p*-value threshold definition, due to the relatively small target sample size. The optimal *p*-value was defined at (*p*_T_)=0.105, *p*=5.36×10^-12^, R^2^= 0.057. The total number of SNPs for BD-PRS at *p*_T_=0.105 was 214,928.

**A)**

**B)**

**
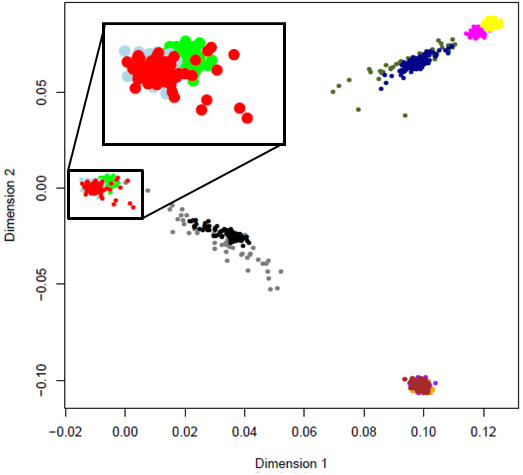
**

**C)**

**
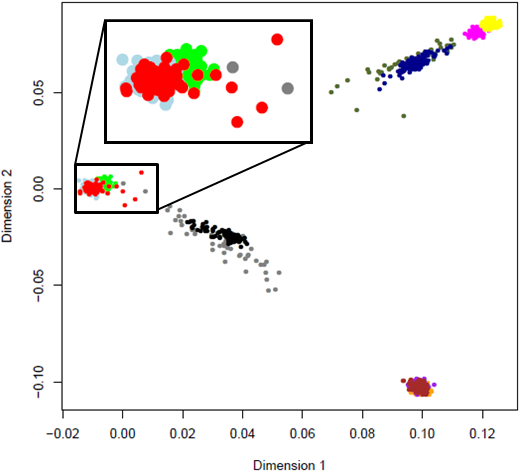
**

**
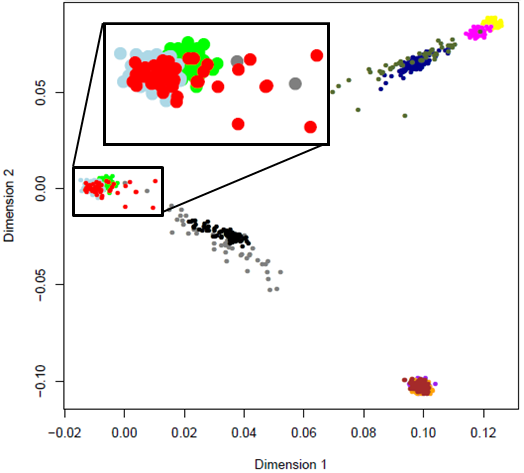
**

**D)**


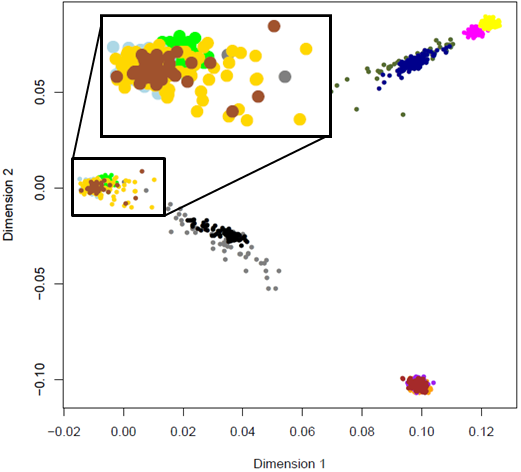


*Figure S2.* Scatter plots of components 1 and 2 from multidimensional scaling analysis, indexing genotype-derived ethnicity of samples employed in the present study as compared to HAPMAP reference populations. A) discovery EWAS, B) validation set 1, C) validation set 2, D) all samples from both discovery and validation sets. Samples from participants analysed in the present study are shown in red (A-C), or are coloured by country of origin (D; Australia=gold, USA=sienna). Individuals from Hapmap reference populations are colour coded as follows: CEU=light blue, TSI=apple green, MEX=grey, GIH=black, CHB=brown, JPT=purple, CHD=orange, YRI=yellow, LWK=magenta, ASW=olive green, MKK=dark blue. *Abbreviations:* ASW, African ancestry in Southwest USA; CEU, Utah residents with Northern and Western European ancestry from the CEPH collection; CHB, Han Chinese in Beijing, China; CHD, Chinese in Metropolitan Denver, Colorado; GIH, Gujarati Indians in Houston, Texas; JPT, Japanese in Tokyo, Japan; LWK, Luhya in Webuye, Kenya; MXL, Mexican ancestry in Los Angeles, California; MKK, Maasai in Kinyawa, Kenya; TSI, Toscani in Italia; YRI, Yoruba in Ibadan, Nigeria.

**A)**

**
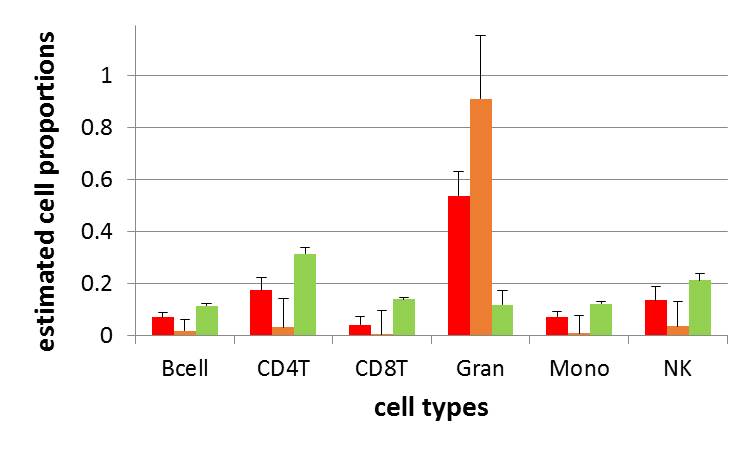
**

**B)**

**
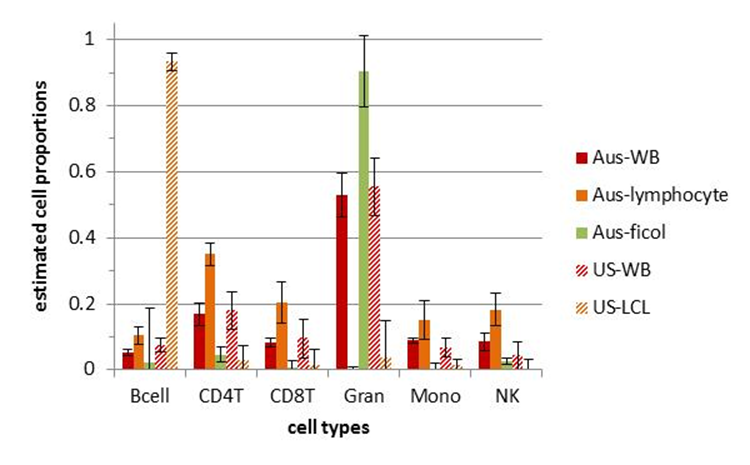
**

*Figure S3.* Estimated blood-cell components for alternative blood tissue sources for Batch 1-3 (panel A) and Batch 4 (panel B) samples. Proportions of six cell-types: B cells (Bcell), CD4 T lymphocytes (CD4T), CD8^+^ T cells (CD8T), granulocytes (gran), monocytes (mono) and natural killer (NK); were estimated performed using *meffil’s* gse35069 profile reference.[23] Proportions are average values that sum to 1, and error bars represent standard deviations. Red bars indicate whole-blood derived samples, orange bars represent lymphocyte-derived samples, green bars indicate ficoll/buffycoat-derived samples; solid colouring indicates Australian participant samples and hatched colouring indicates US participant samples.

1. **B) C)**

*
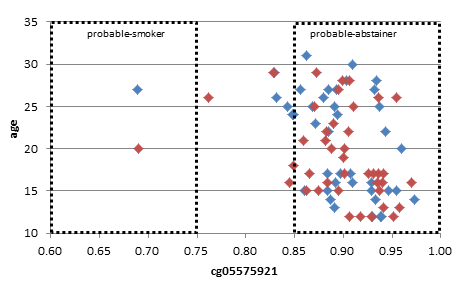

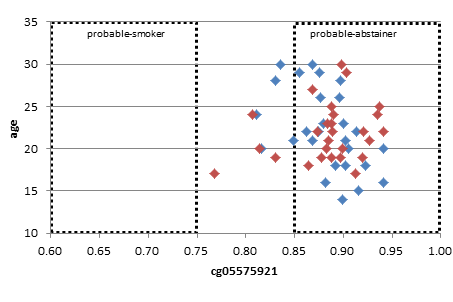

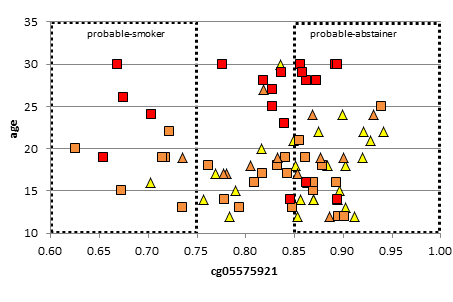
*

*Figure S4.* Predicted smoking status of sample based on methylation status of cg05575921, which lies in an intronic transcription enhancer in the aryl hydrocarbon receptor repressor (AHRR) gene. Individuals with normalised β-values >~0.85 are reported to have >90% probability of abstaining, and individuals with normalised β-values <0.75 are reported to have <~10% probability of abstaining[22]; around 10% of the sample is in the ambiguous range (β=0.75-0.85). Each panel shows a scatter plot of methylation β-values at cg05575921 (x-axis) and baseline age (y-axis). A) EWAS discovery sample of HR participants; *n*=72 (88%) are probable-abstainers, *n*=9 (9.7%) are ambiguous, *n*=2 (2.5%) are probable-smokers. Red diamonds are Low-BD-PRS, blue diamonds are High-BD-PRS. B) validation set 1 (control participants); *n*=45 (83%) are probable-abstainers, *n*=9 (17%) are ambiguous, *n*=0 are probable-smokers. Red diamonds are Low-BD-PRS, blue diamonds are High-BD-PRS. C) validation set 2; baseline group is colour coded as follows: BD=red (*n*=4 probable-smokers), baseline HR=orange (*n*=7 probable-smokers), baseline CON=yellow (*n*=1 probable-smoker). Individuals who were BD-syndromic at follow-up are represented by squares; disorder-free individuals are indicated by triangles.

**
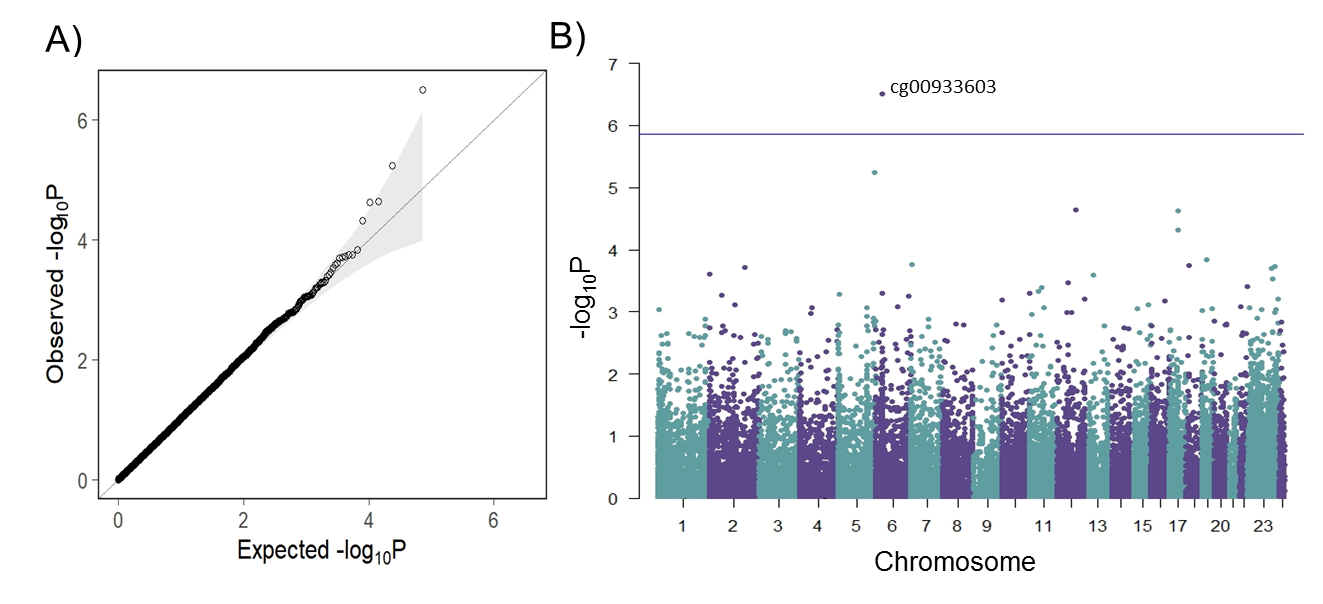
**

*Figure S5.* PRS-stratified EWAS in high-risk participants after correcting for smoking exposure via methylation β-value of cg05575921 in the aryl hydrocarbon receptor repressor (AHRR) gene. A) Quantile-Quantile plot, indicating observed vs. expected *p*-values from 35,907 probes. Bias and inflation corrected to -0.0036 and 0.98, respectively. B) Manhattan plot indicating the genomic location of differentially methylated probes. The vertical line indicates the multiple testing correction threshold at α=0.05 for epigenome-wide association, based on 35,907 probes (*p*<1.39×10^-6^). The location of the top DMP, cg00933603, is indicated. Covariates included age, sex, MDS C1 and C2, tissue source, six blood cell components, four surrogate variables and β_cg05575921_. *Abbreviations:* log, logarithm; P, *p*-value.

**
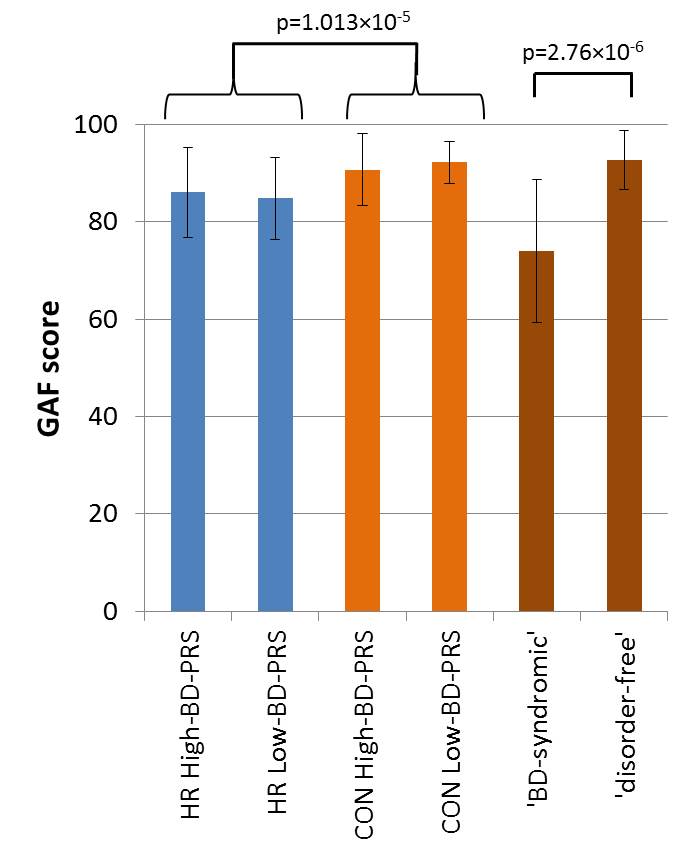
**

*Figure S6.* Global Assessment of Functioning (GAF) rating scales for cohort participants. Mean scores for participants within each subgroup are presented, with error bars indicating standard deviation. Data was available for 198 of 218 participants (91%) across the discovery and validation sets. Scores range from 100 (extremely high functioning) to 1 (severely impaired) – scores of 61-70 indicate some mild symptoms or some difficulty in functioning, and 71-80 indicate transient symptoms and expected reactions to psychosocial stressors. Groups in the discovery EWAS are coloured blue, Validation Set 1 in light orange, Validation Set 2 in dark orange. No difference in GAF score was observed between High-BD-PRS and Low-BD-PRS strata within high risk (*n*=35 vs. 39; *p*=0.54), or control sets (*n*=26 vs. 27; *p*=0.37). GAF score was lower in HR than CON (*n*=74 vs. 53; *M*±*SD*=85.4±8.8 vs. 91.4±6.0, *p*=1.013×10^-5^), and lower in HR High-BD-PRS than CON High-BD-PRS (*n*=35 vs. 26; *M*±*SD*=86.0±9.3 vs. 90.7±7.4; *p*=0.034). Diagnoses within each group are provided in Table S8.


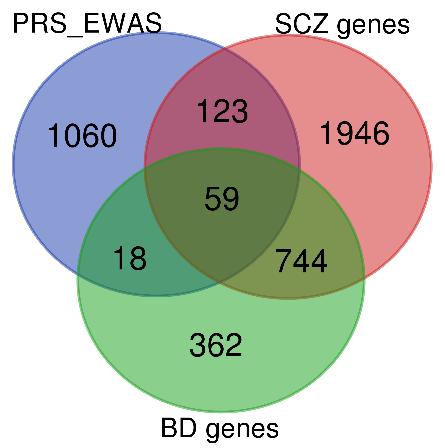


*Figure S7.* Overlap in nominally significant (*p*-value<0.05) differentially methylated genes, with genes reported as associated with BD and SCZ in DisGeNET v7.0.[16]

**
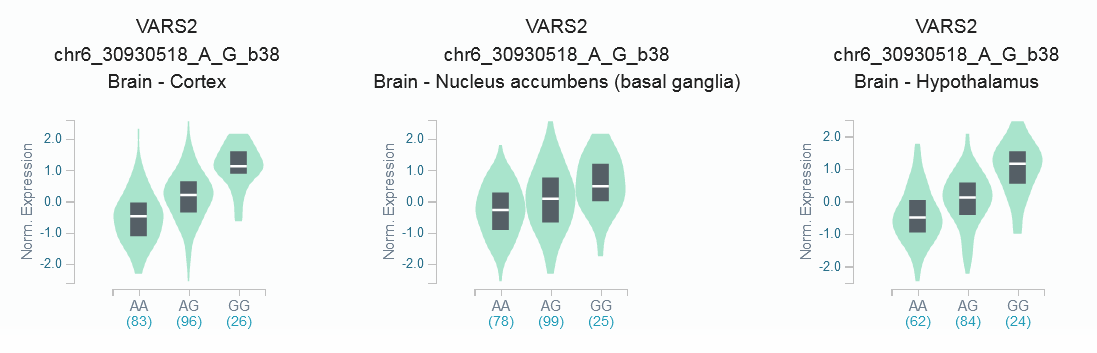
**

*Figure S8.* GTEx gene expression data showing influence of SNP rs2532928 (formerly known as rs116537083) on expression of *VARS2*. The EWAS-significant CpG probe (cg00933603; hg19/chr6: 30,883,002bp) has a replicated *cis*-mQTL SNP rs2532928 (3.133×10^-71^<*p*<8.78×10^-161^, effect range=0.289-0.324).[24] SNP rs2532928 significantly influences *VARS2* gene expression in 16 brain tissues (9.1e^-29^<*p*< 3.1×10^-13^), including cortex (*p*=9.1×10^-29^, normalised effect size=0.73), nucleus accumbens (*p*=3.1×10^-27^, normalised effect size=0.53) and hypothalamus (*p*=3.5×10^-24^, normalised effect size=0.60) (GTEx v8; [25]). SNP rs2532928 (hg19/chr6:30,898,295bp) is not associated with BD in PGC3-BD GWAS (*p*=0.307), but *VARS2* (hg19/chr6:30.88–30.90 Mb) lies in the MHC region (hg19/chr6:25–34 Mb) which shows strong association with BD (max *p*=5.75×10^-15^ at rs13195402, 26.46Mb).[5] *VARS2* is ubiquitously expressed, but with highest expression in the cerebellum.


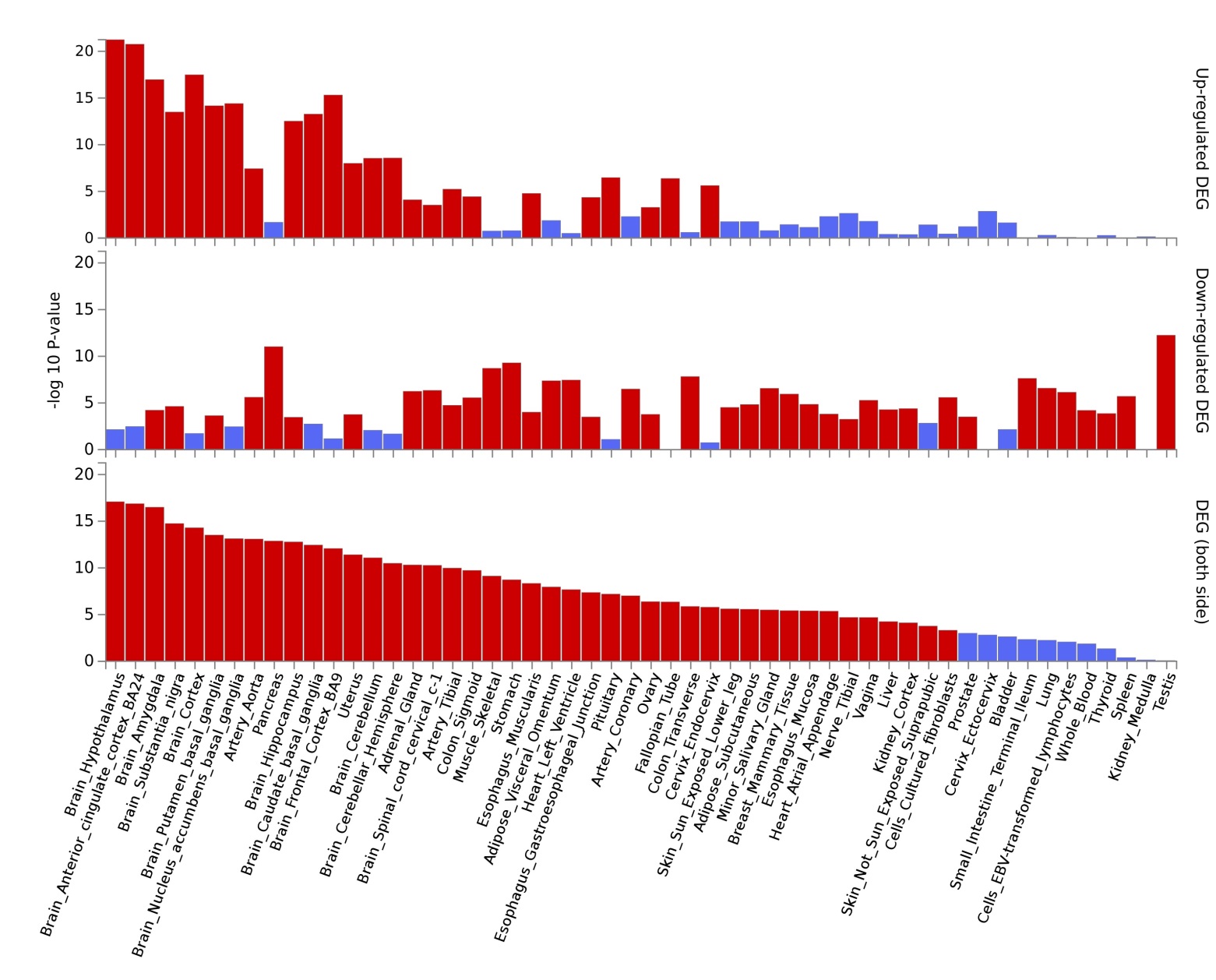


*Figure S9.* Tissue expression analysis of nominal differentially methylated genes (*p*<0.05) from the GTEx v8 database in FUMA [26] analysis of 54 tissue types. Significant enrichment of DMP genes in DEG across tissues is shown in red.


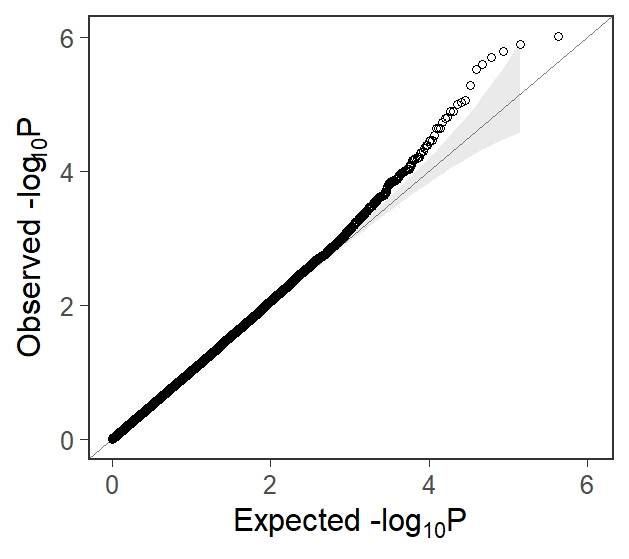


*Figure S10.* Quantile-quantile plot for supplementary EWAS (High vs. Low BD-PRS in HR; *n*=82) for 214,352 probes that passed QC (without applying the brain correlation filter). Analysis employed identical procedures to the primary EWAS after regenerating surrogate variables (*n*=4, based on new probe-set). Plot indicates observed vs. expected *p* with bias and inflation corrected to 0.00097 and 1, respectively. Specific probes that lie above the inflection point (*p*<0.001) are listed in Table S5.

**
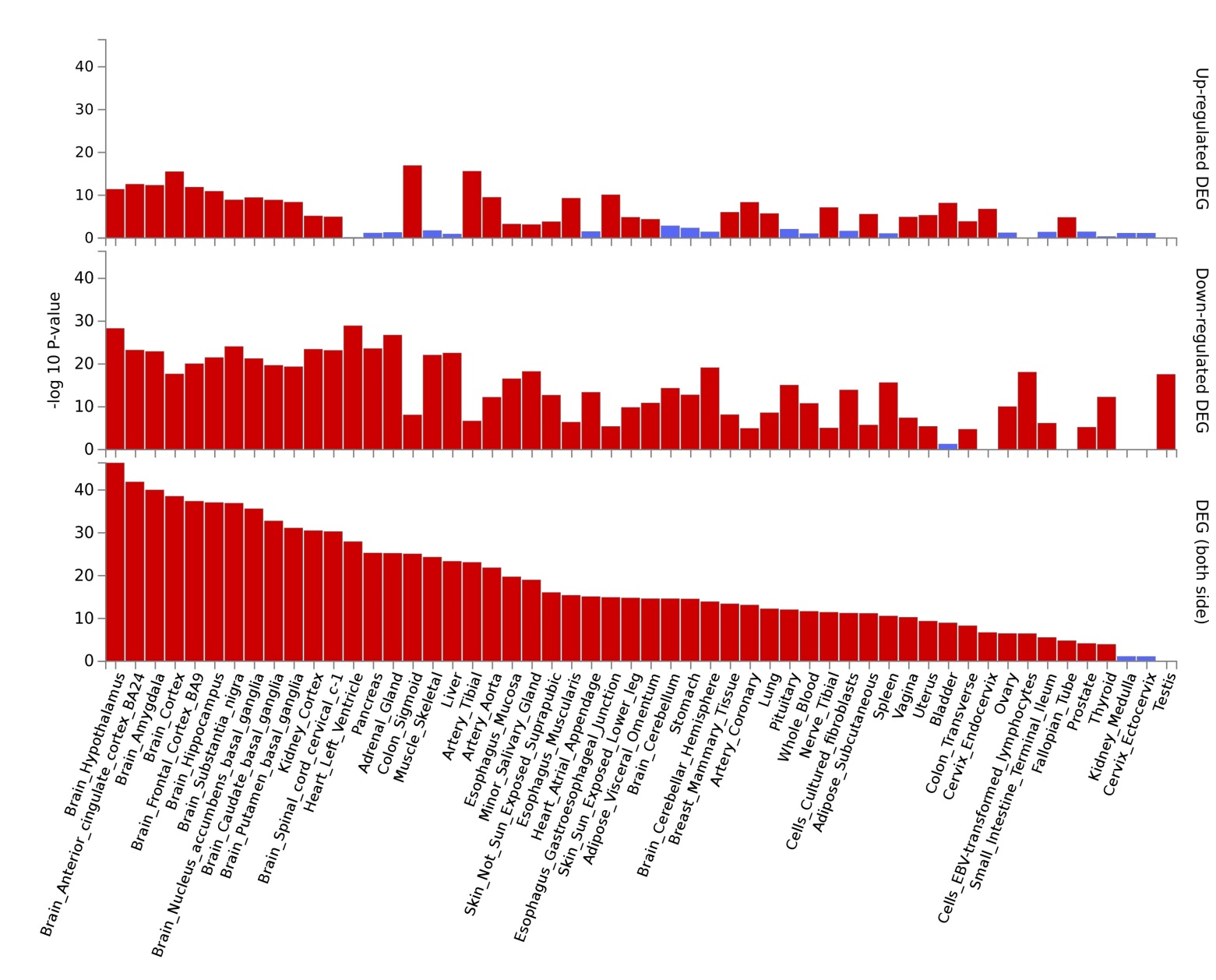
**

*Figure S11.* Tissue expression analysis of 1184 differentially methylated genes (*p*<0.0064) from the supplementary EWAS from the GTEx v8 database in FUMA[26] analysis of 54 tissue types. Significant enrichment of DMP genes in DEG across tissues is shown in red.


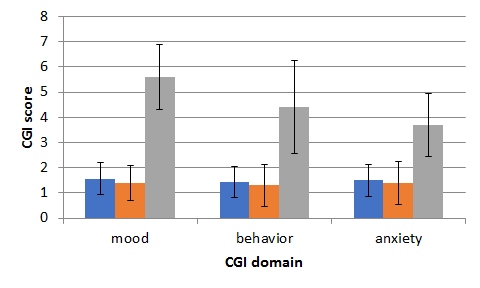


*Figure S12.* Clinical Global Impression (CG)[27] rating scales for US cohort participants in Validation Set 2. Best-estimate diagnoses for HR_unwell_ group (*n*=19; grey) were BD-I/SABP *n*=7, BD-II *n*=7, BD-NOS *n*=6 (one converted between baseline and follow-up assessment). HR_well_ (*n*=9; orange) and CON_well_ (*n*=16; blue) comprised 24 with no diagnosis, and 1 with a non-affective diagnosis. CGI scores are coded as previously described by Busner *et al*:

*1 = Normal — not at all ill, symptoms of disorder not present over past seven days; 2 = Borderline mentally ill — subtle or suspected pathology; 3 = Mildly ill — clearly established symptoms with minimal, if any, distress or difficulty in social and occupational function; 4 = Moderately ill — overt symptoms causing noticeable, but modest, functional impairment or distress with symptom level that may warrant medication; 5 = Markedly ill — intrusive symptoms that distinctly impair social/occupational function or cause intrusive levels of distress; 6 = Severely ill — disruptive pathology, behavior and function are frequently influenced by symptoms, may require assistance from others; 7 = Among the most extremely ill patients — pathology drastically interferes in many life functions and may be hospitalized.*[27]

**Supplementary References**

1. Groen K, Lea RA, Maltby VE, Scott RJ, & Lechner-Scott J. Letter to the editor: blood processing and sample storage have negligible effects on methylation. Clin Epigenetics. 2018; 10:22.

2. Wilcox HC, Fullerton JM, Glowinski AL, Benke K, Kamali M, Hulvershorn LA, et al. Traumatic stress interacts with bipolar disorder genetic risk to increase risk for suicide attempts. J Am Acad Child Adolesc Psychiatry. 2017; 56(12):1073-1080.

3. Purcell S, Neale B, Todd-Brown K, Thomas L, Ferreira MA, Bender D, et al. PLINK: a tool set for whole-genome association and population-based linkage analyses. Am J Hum Genet. 2007; 81(3):559-575.

4. Das S, Forer L, Schönherr S, Sidore C, Locke AE, Kwong A, et al. Next-generation genotype imputation service and methods. Nat Genet. 2016; 48(10):1284-1287.

5. Mullins N, Forstner AJ, O’Connell KS, Coombes B, Coleman JR, Qiao Z, et al. Genome-wide association study of more than 40,000 bipolar disorder cases provides new insights into the underlying biology. Nat Genet. 2021:1-13.

6. Nurnberger JI, McInnis M, Reich W, Kastelic E, Wilcox HC, Glowinski A, et al. A high-risk study of bipolar disorder: childhood clinical phenotypes as precursors of major mood disorders. Arch Gen Psychiatry. 2011; 68(10):1012-1020.

7. Watkeys OJ, Cohen-Woods S, Quidé Y, Cairns MJ, Overs B, Fullerton JM, et al. Derivation of poly-methylomic profile scores for schizophrenia. Prog Neuropsychopharmacol Biol Psychiatry. 2020; 101:109925.

8. Mitchell PB, Johnston AK, Corry J, Ball JR, Malhi GSJA, & Psychiatry NZJo. Characteristics of bipolar disorder in an Australian specialist outpatient clinic: comparison across large datasets. Aust N Z J Psychiatry. 2009; 43(2):109-117.

9. American Psychiatric Association, Diagnostic and statistical manual of mental disorders. 4th ed. (DSM-IV) ed. American Psychiatric Association. 1994, Washington D.C.: American Psychiatric Press Inc.

10. Nurnberger JI, Jr., Blehar MC, Kaufmann CA, York-Cooler C, Simpson SG, Harkavy-Friedman J, et al. Diagnostic interview for genetic studies. Rationale, unique features, and training. NIMH Genetics Initiative. Arch Gen Psychiatry. 1994; 51(11):849-59; discussion 863-4.

11. Maxwell ME. Family Interview for Genetic Studies (FIGS): a manual for FIGS. Clinical Neurogenetics Branch, National Institute of Mental Health. 1992.

12. Williams JB, Gibbon M, First MB, Spitzer RL, Davies M, Borus J, et al. The Structured Clinical Interview for DSM-III-R (SCID). II. Multisite test-retest reliability. Arch Gen Psychiatry. 1992; 49(8):630-6.

13. Gatt JM, Korgaonkar MS, Schofield PR, Harris A, Clark CR, Oakley KL, et al. The TWIN-E project in emotional wellbeing: study protocol and preliminary heritability results across four MRI and DTI measures. Twin Res Hum Genet. 2012; 15(3):419-41.

14. Jamshidi J, Williams LM, Schofield PR, Park HR, Montalto A, Chilver MR, et al. Diverse phenotypic measurements of wellbeing: Heritability, temporal stability and the variance explained by polygenic scores. Genes Brain Behav. 2020; 19(8):e12694.

15. Price AL, Patterson NJ, Plenge RM, Weinblatt ME, Shadick NA, & Reich D. Principal components analysis corrects for stratification in genome-wide association studies. Nat Genet. 2006; 38(8):904-9.

16. Piñero J, Ramírez-Anguita JM, Saüch-Pitarch J, Ronzano F, Centeno E, Sanz F, et al. The DisGeNET knowledge platform for disease genomics: 2019 update. Nucleic Acids Res. 2020; 48(D1):D845-D855.

17. Gutiérrez-Sacristán A, Grosdidier S, Valverde O, Torrens M, Bravo À, Piñero J, et al. PsyGeNET: a knowledge platform on psychiatric disorders and their genes. Bioinformatics. 2015; 31(18):3075-7.

18. MacArthur J, Bowler E, Cerezo M, Gil L, Hall P, Hastings E, et al. The new NHGRI-EBI Catalog of published genome-wide association studies (GWAS Catalog). Nucleic Acids Res. 2017; 45(D1):D896-d901.

19. Li MJ, Liu Z, Wang P, Wong MP, Nelson MR, Kocher JP, et al. GWASdb v2: an update database for human genetic variants identified by genome-wide association studies. Nucleic Acids Res. 2016; 44(D1):D869-76.

20. Olson DH, Portner J, & Bell RQ. FACES II: Family Adaptability and Cohesion Evaluation Scale. St. Paul: University of Minnesota, Department of Family Social Science. 1982.

21. Saffari A, Silver MJ, Zavattari P, Moi L, Columbano A, Meaburn EL, et al. Estimation of a significance threshold for epigenome-wide association studies. Genet Epidemiol. 2018; 42(1):20-33.

22. Dawes K, Andersen A, Reimer R, Mills JA, Hoffman E, Long JD, et al. The relationship of smoking to cg05575921 methylation in blood and saliva DNA samples from several studies. Sci Rep. 2021; 11(1):21627.

23. Min JL, Hemani G, Davey Smith G, Relton C, & Suderman M. Meffil: efficient normalization and analysis of very large DNA methylation datasets. Bioinformatics. 2018; 34(23):3983-3989.

24. McRae AF, Marioni RE, Shah S, Yang J, Powell JE, Harris SE, et al. Identification of 55,000 Replicated DNA Methylation QTL. Sci Rep. 2018; 8(1):17605.

25. GTEx Consortium. The Genotype-Tissue Expression (GTEx) project. Nat Genet. 2013; 45(6):580-5.

26. Watanabe K, Taskesen E, Van Bochoven A, & Posthuma D. Functional mapping and annotation of genetic associations with FUMA. Nat Commun. 2017; 8(1):1-11.

27. Busner J & Targum SD. The clinical global impressions scale: applying a research tool in clinical practice. Psychiatry (Edgmont). 2007; 4(7):28-37.
